# Systematic Metabolic Profiling Identifies *De Novo* Sphingolipid Synthesis as Hypha Associated and Essential for Candida albicans Filamentation

**DOI:** 10.1128/msystems.00539-22

**Published:** 2022-10-20

**Authors:** Enrico Garbe, Franziska Gerwien, Dominik Driesch, Tina Müller, Bettina Böttcher, Markus Gräler, Slavena Vylkova

**Affiliations:** a Septomics Research Center, Friedrich Schiller University and Leibniz Institute for Natural Product Research and Infection Biology—Hans Knöll Institute, Jena, Germany; b BioControl Jena, Jena, Germany; c Department of Anesthesiology and Intensive Care Medicine, Center for Molecular Biomedicine (CMB) and Center for Sepsis Control and Care (CSCC), Jena University Hospital, Jena, Germany; Max Planck Institute for Marine Microbiology

**Keywords:** *Candida albicans*, filamentation, metabolomics, metabolic adaptation, sphingolipids, hyphal development, sphingolipid biosynthesis

## Abstract

The yeast-to-hypha transition is a key virulence attribute of the opportunistic human fungal pathogen Candida albicans, since it is closely tied to infection-associated processes such as tissue invasion and escape from phagocytes. While the nature of hypha-associated gene expression required for fungal virulence has been thoroughly investigated, potential morphotype-dependent activity of metabolic pathways remained unclear. Here, we combined global transcriptome and metabolome analyses for the wild-type SC5314 and the hypha-defective *hgc1*Δ and *cph1*Δ*efg1*Δ strains under three hypha-inducing (human serum, *N*-acetylglucosamine, and alkaline pH) and two yeast-promoting conditions to identify metabolic adaptions that accompany the filamentation process. We identified morphotype-related activities of distinct pathways and a metabolic core signature of 26 metabolites with consistent depletion or enrichment during the yeast-to-hypha transition. Most strikingly, we found a hypha-associated activation of *de novo* sphingolipid biosynthesis, indicating a connection of this pathway and filamentous growth. Consequently, pharmacological inhibition of this partially fungus-specific pathway resulted in strongly impaired filamentation, verifying the necessity of *de novo* sphingolipid biosynthesis for proper hypha formation.

**IMPORTANCE** The reversible switch of Candida albicans between unicellular yeast and multicellular hyphal growth is accompanied by a well-studied hypha-associated gene expression, encoding virulence factors like adhesins, toxins, or nutrient scavengers. The investigation of this gene expression consequently led to fundamental insights into the pathogenesis of this fungus. In this study, we applied this concept to hypha-associated metabolic adaptations and identified morphotype-dependent activities of distinct pathways and a stimulus-independent metabolic signature of hyphae. Most strikingly, we found the induction of *de novo* sphingolipid biosynthesis as hypha associated and essential for the filamentation of C. albicans. These findings verified the presence of morphotype-specific metabolic traits in the fungus, which appear connected to the fungal virulence. Furthermore, the here-provided comprehensive description of the fungal metabolome will help to foster future research and lead to a better understanding of fungal physiology.

## INTRODUCTION

Cellular metabolism, the entirety of all biochemical reactions in a living cell, is a highly complex and interconnected system, which provides the basis for growth, cellular maintenance, and responses to changing environments and therefore the foundation of life. Although the main branches of primary metabolism, comprising the central carbon and nitrogen metabolism, are evolutionarily conserved and remarkably stable among all organisms, significant differences are found between individual phyla and clades (1). The fungus Candida albicans, like many other opportunistic pathogens, is especially challenged by the ever-changing environments of its respective host niches, which are often limited in nutrients (2, 3). Thus, metabolic plasticity is central for its host-associated lifestyle and consequently viewed as one of its main virulence factors (4–7). The fungus is able to utilize a variety of different nutrient sources, like sugars, lipids, and carboxylic and amino acids, and therefore possesses a plethora of sophisticated sensing and uptake mechanisms (8–14). In this regard, unique metabolic features, modified or absent in the host, are of special interest in the development of novel therapeutic strategies against *Candida* infections (15).

Besides its highly adaptive metabolism, arguably the most acknowledged virulence factor of C. albicans is its polymorphism and especially the ability to undergo a reversible yeast-to-hypha transition (4, 16). Over the past decades the underlying physiology, regulation, stimuli, and function of hyphae were subjected to extensive investigation, particularly in the context of host-pathogen interactions (16–20). Several transcriptional studies specifically detailed gene expression changes during filamentation and identified key genes associated with hyphae (21–26). Interestingly, many of the known hyphal stimuli represent metabolic cues, like nitrogen starvation, low sugar levels, exposure to serum components, or alternative carbon sources such as proline or *N*-acetylglucosamine (GlcNAc), indicating a close connection between cellular metabolism and morphogenic state (27–31). Yet, the changes in cellular metabolism that accompany the yeast-to-hypha transition are far less known.

Metabolomics emerged as a versatile tool to investigate the precise metabolic state of the cell (32). Few recent studies have since focused on metabolic changes and adaptation processes in C. albicans (32–37). One previous study suggested that hyphae represent an energy-conserving state and a general shutdown of the central carbon metabolism in fungal cells undergoing filamentation (35). However, compared to recently published metabolomes in this study only a limited number of metabolites was detected, which therefore might provide an incomplete picture (36, 37).

Within our study we combined transcriptomics and metabolomics for the C. albicans wild-type strain SC5314 (WT) and two filamentation-impaired mutant strains—the *cph1*Δ*efg1*Δ and *hgc1*Δ strains (38–42)—grown under different yeast- or hypha-inducing conditions to address the following questions. (i) Are hyphae indeed metabolically less active? (ii) Is the hyphal state characterized by a stimulus-independent metabolic core response? (iii) Can potential morphotype-dependent metabolic features be found in nonfilamentous mutants? (iv) Do the transcriptome and metabolome of the cell align, and can the former be used to predict the metabolic state of the cell?

Overall, we verified and extended previous findings of an inducer-independent core set of morphotype-associated genes (21). Only some of those were clearly associated with metabolism, and we further found little overlay of transcriptional and metabolic profiles. On the metabolic side, we detected 638 metabolites with no evidence supporting the proposed shutdown of the central carbon metabolism in hyphae compared to yeast (35). Moreover, we identified a hypha-specific metabolic core signature, comprising a set of 26 metabolites with differential abundance in hyphae compared to yeast.

Most striking was the consistently reduced abundance of the first three consecutive intermediates of *de novo* sphingolipid biosynthesis in this group, indicating a connection between this pathway and hypha formation in C. albicans. Indeed, we observed a block in filamentation upon application of sublethal concentrations of sphingolipid biosynthesis inhibitors under otherwise hypha-inducing conditions. Notably, the addition of the downstream metabolite phytosphingosine restored hypha formation under conditions where the first step of the biosynthetic pathway was inhibited. Phytosphingosine is a precursor for the synthesis of acidic glycosphingolipids (GSLs), which indicates a striking role of this metabolite class for filamentation. Since their synthesis is fungus specific, it represents a promising target for antifungal therapy. Summarized, our findings comprehensively describe the fungal metabolome in response to hypha-inducing stimuli and identified *de novo* sphingolipid biosynthesis as an essential prerequisite of C. albicans cells undergoing the yeast-to-hypha transition.

## RESULTS

### Setup for transcriptional and metabolic profiling under yeast and hyphal-growth conditions.

**FIG 1:**
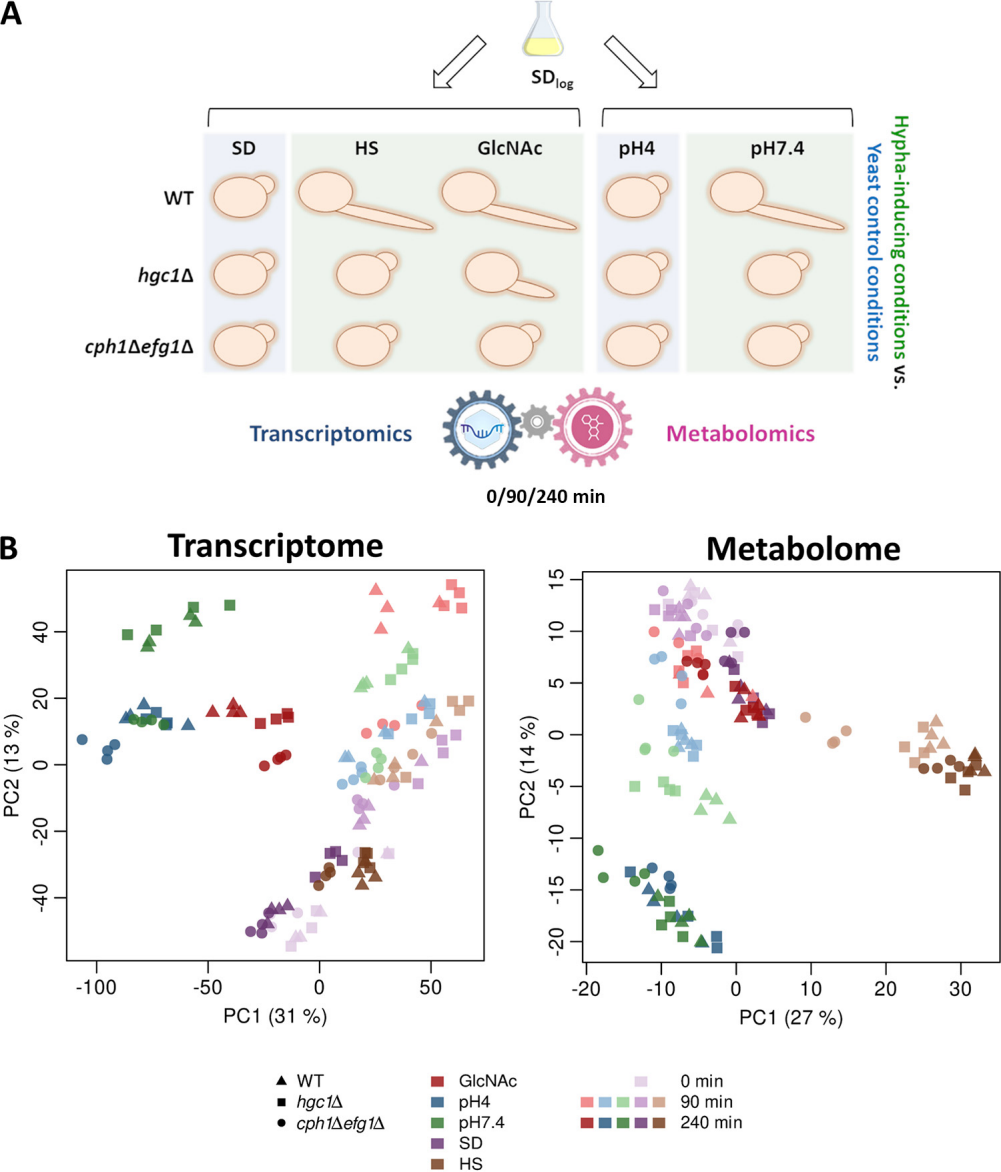
Overview of metabolic and transcriptional profiling during yeast-to-hypha transition. (A) Study setup: C. albicans WT, *hgc1*Δ, and *cph1*Δ *efg1*Δ strains were grown in SD medium until early log phase and transferred to yeast-inducing (SD, pH 4) and hypha-inducing (GlcNAc, HS, and pH 7.4) media. Samples for transcriptional and metabolic profiling were taken simultaneously from identical cultures in four biological replicates. Sampling time points were after 0 (SD log phase only), 90, and 240 min. To identify hypha-associated changes, conditions were always compared as follows: HS and GlcNAc versus SD and pH 7.4 versus pH 4. (B) PCA plots reveal differing clustering behaviors between transcriptome and metabolome. Each symbol represents a biological replicate belonging to a test condition characterized by three parameters: strain (shape), medium type (color), and time point (color intensity). For example, dark red triangles represent replicates for WT in GlcNAc at 240 min.

Hyphal stimuli are often directly related to metabolism, which aggravates the discrimination between medium- or hypha-caused effects when investigating the hyphal metabolome. Therefore, to minimize medium-derived effects, we compared three different, well-established hypha-inducing conditions, M199 pH 7.4 (pH7.4), synthetic dextrose medium (SD) with 10% human serum (HS), and GlcNAc, with two respective yeast growth conditions, M199 pH 4 (pH4) and SD (Fig. 1A). Samples for transcriptional and metabolic profiling were taken at *t* = 0 (SD log phase), 90 min (germ tube formation), and 240 min (hyphal elongation). To identify possible connections between the fungal metabolome and morphotype, we used two filamentation-impaired strains in addition to the WT: the *cph1*Δ *efg1*Δ strain, lacking two central transcription factors required for filamentation (38–40), and the *hgc1*Δ strain, a deletion strain lacking the downstream effector and cyclin Hgc1, which is impaired in filamentation but possesses unaffected expression of hypha-associated genes (41, 43).

As expected, the WT formed hyphae in HS, GlcNAc, and pH 7.4 and remained in the yeast form in SD and pH 4, while the *cph1*Δ *efg1*Δ strain exclusively displayed yeast growth under all conditions (see Fig. S1A in the supplemental material). Similarly, the *hgc1*Δ strain was afilamentous under all conditions but GlcNAc, where filamentation was unexpectedly initiated, although the hyphae were significantly shorter than those of the WT (Fig. S1B). This observation indicates an as-yet-unknown way of Hgc1-independent filamentation and was very recently verified in another study (44).

10.1128/msystems.00539-22.1 FIG S1 C. albicans WT and mutant filamentation characteristics in the settings used for this study. (A) Microscopic overview of cell morphology under all tested conditions at 90 and 240 min and SD log phase. Filamentation score: 0, yeast; +, germ tubes and small hyphae; ++, moderate filamentation; +++, extensive filamentation. Bar, 50 μm. (B) Quantification of hyphal length reveals shorter hyphae for the *hgc1*Δ strain than for the WT in GlcNAc after 240 min. *n* = 3. For each replicate 100 hyphae were measured. Unpaired *t* test, Welch’s correction; ****, *P* < 0.0001; dashed lines, median; lower dotted lines, 25th percentile; upper dotted lines, 75th percentile. Download FIG S1, TIF file, 1.5 MB. Copyright © 2022 Garbe et al. 2022 Garbe et al. https://creativecommons.org/licenses/by/4.0/ This content is distributed under the terms of the Creative Commons Attribution 4.0 International license.

### Transcriptome and metabolome show no clear morphotype-dependent correlation.

The obtained samples were processed for transcriptional profiling via RNA sequencing and metabolic profiling via mass spectrometry (MS). At first, we performed a principal-component analysis (PCA) with both data sets to compare the clustering of the individual groups. Overall, the single replicates of each sample group clustered together, indicating a high reliability of the data (Fig. 1B).

For the transcriptome, clear time-dependent effects were visible with different time points clustering apart from each other, indicating vast transcriptional changes over the onset of filamentation as well as during medium adaption (Fig. 1B). Additionally, the WT and *hgc1*Δ strains clustered mostly together, indicating similar gene expression despite partially differing morphotypes. Notably, for both GlcNAc time points a slight separation was visible—potentially associated with compensatory effects in the *hgc1*Δ strain, which are involved in its partially retained ability to filament in GlcNAc. The *cph1*Δ *efg1*Δ mutant appeared transcriptionally distinct from the other strains, especially under hypha-inducing conditions—for example, the pH 7.4 samples clustered together with the pH 4 yeast control replicates for both time points. Interestingly, no clear separation could be observed for the *hgc1*Δ strain between the SD and HS replicates, while the WT and *cph1*Δ *efg1*Δ strains formed medium-specific clusters at 240 min.

The PCA of the metabolome revealed a different distribution—for instance, all GlcNAc and SD samples clustered together, despite the clear separation in the transcriptome and various morphotypes (Fig. 1B). Furthermore, all M199 samples formed two distinct clusters, separated by time and independent of genotype and morphotype. While more similar on the transcriptional level, all serum samples clearly separated from the SD medium and formed an individual cluster.

In summary, the transcriptome clustered according to almost all parameters, by time point, medium, and genotype, with only partially apparent morphotype-related effects. However, the clustering of the metabolome was distinct and dominated by medium and specific time point effects, showing only little, if any, influence of genotype or morphotype.

### Transcriptional profiling reveals an expanded morphotype-associated gene expression.

Next, we aimed to determine the transcriptional core response of the WT consisting of consistently up- or downregulated genes during filamentation. Therefore, we compared hypha-inducing with yeast-promoting growth conditions as follows: HS and GlcNAc versus SD and pH 7.4 versus pH 4. The complete expression data and calculated fold changes are provided as “Transcriptomics results” at https://doi.org/10.5281/zenodo.7019410.

**FIG 2:**
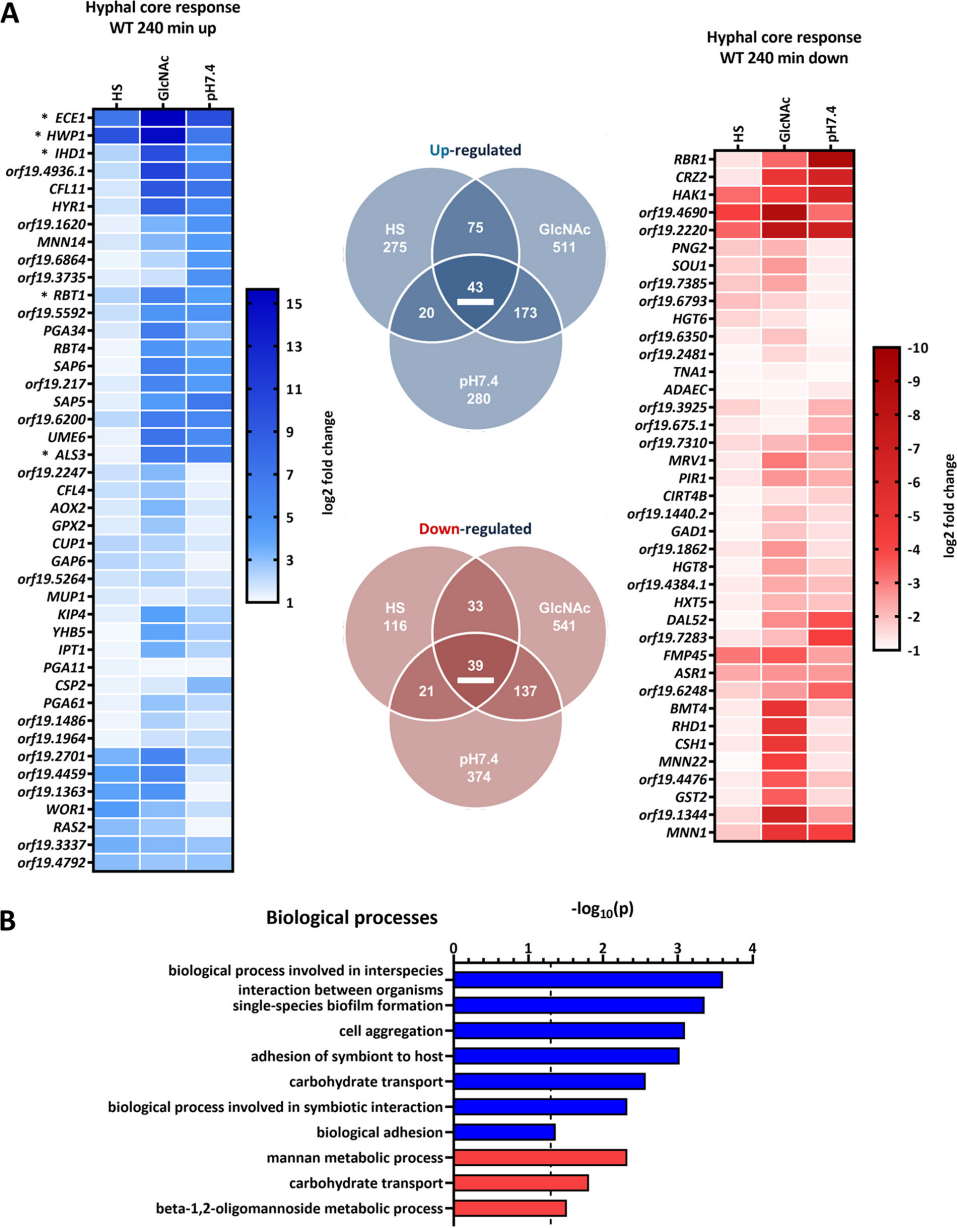
Transcriptional core response of WT hyphae after 240 min. (A) Genes affected by the yeast-to-hypha transition in elongated hyphae (HS and GlcNAc versus SD and pH 7.4 versus pH 4) after 240 min. Only genes meeting the following criteria were included: *P* < 0.05 and log_2_ fold change ≤−1 or ≥1. Upregulation is indicated in blue and downregulation in red. Expression changes (log_2_) of the respective core response genes (underlined in the Venn diagram) are displayed. Asterisks indicate core filamentation response genes previously described in the work of Martin et al. (21). (B) Significantly enriched GO terms for the hyphal core response in the WT at 240 min. GO analysis was performed twice with either upregulated (blue) or downregulated (red) genes. Significance threshold for GO terms [*P* = −log_10_(0.05)] is indicated by the dashed line.

In total, we found 82 genes to be consistently, differentially expressed (43 upregulated, 39 downregulated) in elongated hyphae (240 min) (Fig. 2A). The upregulated set included five (*ECE1*, *HWP1*, *ALS3*, *IHD1*, and *RBT1*) of eight genes from the previously described C. albicans “core filamentation response” set from the work of Martin et al. (21). Another two genes (*DCK1* and *orf19.2457*) met our cutoff criteria only for the 90 min time point, while the last gene (*HGT2*) was not significantly altered in our settings. Notably, the core set of upregulated genes for the 90 min time point was substantially larger (Fig. S2A). These changes could mostly be attributed to higher expression levels of hypha-associated genes in HS after 90 min than after 240 min, indicating a quicker and swiftly abated response for HS samples (Fig. S2B). Yet, this appeared specific for these hypha-associated genes, as the overall number of differentially expressed genes in HS increased from 90 to 240 min (Fig. 2A and Fig. S2A). Generally, only a partial overlap of the core sets for germinating and elongated hyphae was observed, indicating an additional time point-specific response (Fig. S2B). Nevertheless, our results significantly increase the number of genes commonly affected by the yeast-to-hypha transition, especially interesting in terms of the consistently downregulated genes, a so-far-understudied group.

10.1128/msystems.00539-22.2 FIG S2 Comparison of WT transcriptional core sets after 90 and 240 min of hypha induction. (A) WT hyphal core response (HS and GlcNAc versus SD, pH 7.4 versus pH 4) at 90 min. Only genes meeting the following criteria were included: *P* of <0.05 and log_2_ fold change of ≤−1 or ≥1. Upregulation is indicated in blue, and downregulation is in red. Expression changes (log_2_) of the respective core response genes (underlined in the Venn diagram) are displayed. Asterisks indicate core filamentation response genes previously described in the work of Martin et al. (21). (B) Comparison of the transcriptional core sets for 90 and 240 min. Expression changes (log_2_) of the genes consistently affected in both sets are displayed. Download FIG S2, TIF file, 0.9 MB. Copyright © 2022 Garbe et al. 2022 Garbe et al. https://creativecommons.org/licenses/by/4.0/ This content is distributed under the terms of the Creative Commons Attribution 4.0 International license.

To identify potential processes generally affected by hyphal transition or in response to single stimuli, we next performed a gene set enrichment analysis via gene ontology (GO). Aside from the expected GO terms associated with filamentation and adhesion, “carbohydrate transport” appeared as a conserved and metabolism-related feature of hyphae as it was enriched in both categories—up- and downregulated genes (Fig. 2B). The transcriptional response to the single hyphal stimuli displayed a greater variety of affected terms, including metabolism-associated terms, pointing to medium-specific adaptation (Table S1).

10.1128/msystems.00539-22.7 TABLE S1 GO analysis. Download Table S1, XLSX file, 0.1 MB. Copyright © 2022 Garbe et al. 2022 Garbe et al. https://creativecommons.org/licenses/by/4.0/ This content is distributed under the terms of the Creative Commons Attribution 4.0 International license.

The transcriptional responses of the two mutant strains to hypha-inducing media differed substantially. Compared to the WT, the *hgc1*Δ strain displayed notable medium-specific gene expression differences, while only 12 genes were consistently differentially expressed, confirming the present WT-like hypha-associated gene expression (Fig. S3A). Comparatively, the *cph1*Δ *efg1*Δ strain displayed substantially greater changes (Fig. S3B). In addition to medium-specific differences, we identified a core set of genes differentially expressed in the *cph1*Δ *efg1*Δ strain in hypha-inducing media. This set resembled the mutants’ nonfilamentous state, since various genes associated with hyphal growth in the WT strain were consistently less expressed in the *cph1*Δ *efg1*Δ strain.

10.1128/msystems.00539-22.3 FIG S3 Transcriptional core difference between filamentation-impaired strains and the WT. Differentially expressed genes (*P* of <0.05 and log_2_ fold change of ≤−1 or ≥1) in the three hypha-inducing media (HS, GlcNAc, and pH 7.4) in the indicated comparisons at 240 min: *hgc1*Δ strain versus WT (A) and *cph1*Δ *efg1*Δ strain versus WT (B). Upregulation is indicated in blue, and downregulation is indicated in red. Expression changes (log_2_) of the respective core response genes (underlined in the Venn diagram) are displayed. Asterisks indicate WT hypha-associated genes at 240 min, consistently less expressed in the *cph1*Δ *efg1*Δ strain than in the WT. Download FIG S3, TIF file, 1.0 MB. Copyright © 2022 Garbe et al. 2022 Garbe et al. https://creativecommons.org/licenses/by/4.0/ This content is distributed under the terms of the Creative Commons Attribution 4.0 International license.

### Metabolic profiling of C. albicans hyphae.

** (i) Untargeted global metabolomics provide a comprehensive description of the fungal metabolome.** To obtain a thorough overview of the fungal metabolome upon hyphal transition, we performed untargeted global metabolomics under the abovementioned conditions. The metabolic profiling was performed by Metabolon (Durham, NC, USA). In total, 638 metabolites were detected within the scope of this study. For subsequent analyses each metabolite was unambiguously assigned to one category of a “superpathway” and a secondary “subpathway” (see “Metabolomics results” at https://doi.org/10.5281/zenodo.7019410).

**FIG 3:**
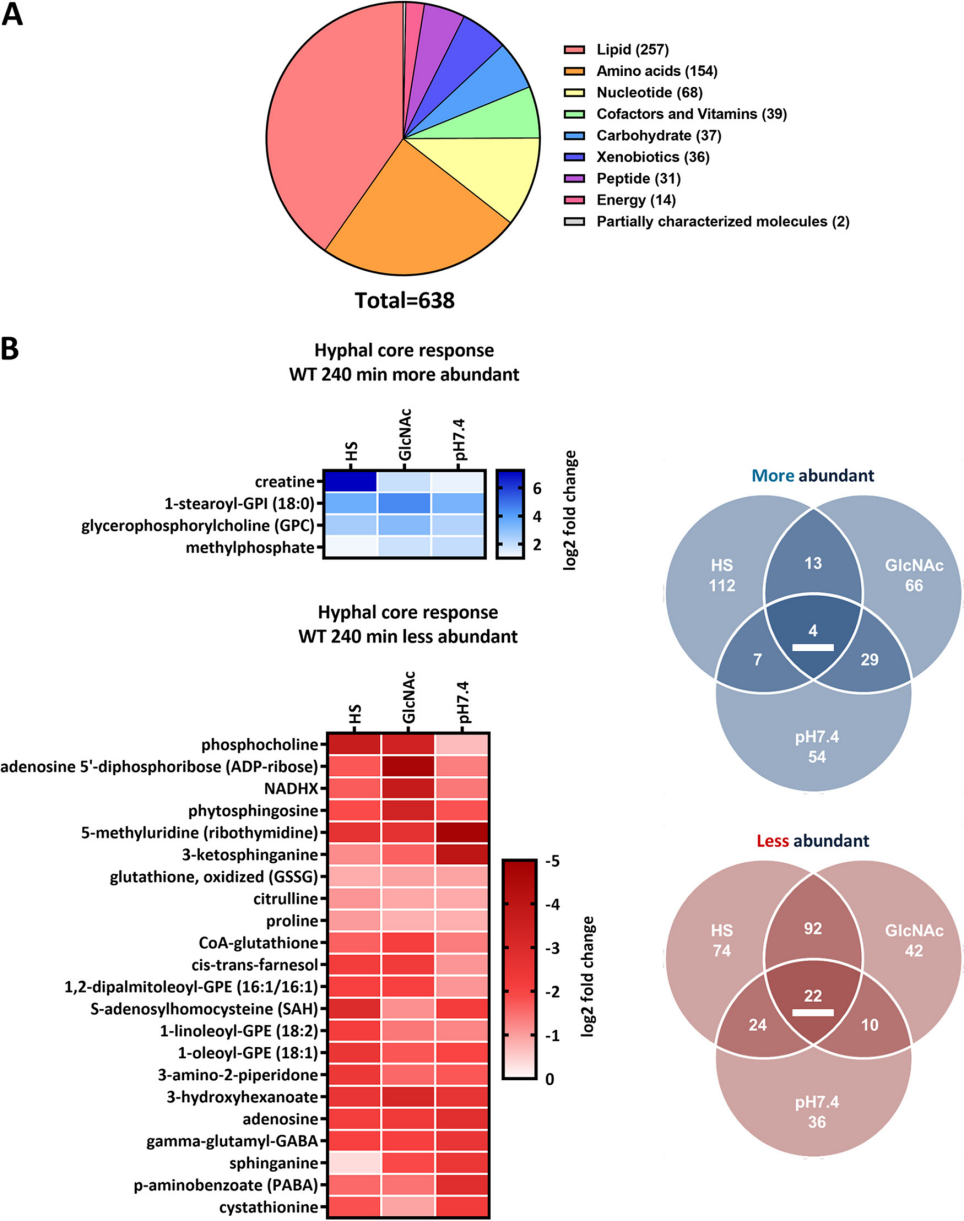
Metabolite categorization and metabolic core response of WT hyphae after 240 min. (A) Metabolites detected in this study grouped by their respective “superpathways.” (B) Metabolites affected by the yeast-to-hypha transition in elongated hyphae (HS and GlcNAc versus SD, pH 7.4 versus pH 4) after 240 min. Only metabolites meeting the threshold of a *P* value of <0.05 are displayed. More abundant metabolites are indicated in blue, and less abundant metabolites are indicated in red. Abundance changes (log_2_) of the respective core set metabolites (underlined in the Venn diagram) are displayed.

Out of all detected metabolites the largest fraction was assigned to the superpathway “Lipids” (~40%), while the next groups according to size were “Amino acids” and “Nucleotides” (24% and 10%, respectively) (Fig. 3A). From the 638 identified metabolites, 529 had a Human Metabolome Database (HMDB) identifier and were used for enrichment analyses via “MetaboAnalyst” (45). Metabolites lacking an HMDB identifier predominantly belonged to the “Lipids” superpathway. Of note, we subsequently used solely a *P*-value-based cutoff for the analysis of metabolite abundance changes to avoid an otherwise arbitrary classification of the level at which metabolite abundance changes become biologically relevant.

First, we visualized the relative abundance of the detected metabolites, clustered by sample and within their respective superpathways. Overall, the clustering verified the observations from the PCA (Fig. 1B), as medium and time point dominated the clustering without apparent morpho- or genotypic influence (Fig. S4A). However, we observed a highly increased diversity of lipids in the HS samples, likely originating from attached or utilized serum components, since human-specific compounds (e.g., sphingomyelins) were detected and the lipid variety in the other samples was markedly lower (Fig. S4B and “Metabolomics results” at https://doi.org/10.5281/zenodo.7019410).

10.1128/msystems.00539-22.4 FIG S4 Z score-based heatmaps of metabolite abundances. Symbols indicate sample groups by the following characteristics: strain (shape), medium type (color), and time point (color intensity). Z scores of each metabolite in the respective conditions are presented for all metabolites detected in this study grouped by their respective superpathways (A) (the dendrogram provides information about distances between single conditions and clusters) and metabolites from the lipid superpathway grouped by their respective subpathways (B). Comparable to results in the PCA, a general clustering according to culture medium and time point is visible, except for GlcNAc and SD (0 and 90 min). WT and *hgc1*Δ strain metabolic compositions appear similar, while the *cph1*Δ *efg1*Δ strain appears distinct from those two strains. Notably, HS shows the exclusive presence of additional lipid classes, like sphingomyelins, likely originating from the human serum. Download FIG S4, TIF file, 0.9 MB. Copyright © 2022 Garbe et al. 2022 Garbe et al. https://creativecommons.org/licenses/by/4.0/ This content is distributed under the terms of the Creative Commons Attribution 4.0 International license.

**(ii) Metabolic profiling reveals a metabolic core signature of hyphae.** Next, analogous to the transcriptional data set, we aimed to define the core set of metabolites always affected by the yeast-to-hypha transition. Overall, a notable number of metabolites was significantly altered after 240 min across the three hypha-inducing conditions for the WT. There were, however, more metabolites with a significantly altered abundance in HS and GlcNAc than in pH 7.4 (Fig. 3B). We could identify a core set of 4 consistently enriched and 22 depleted metabolites, ranging from nucleotide synthesis intermediates, vitamins, and amino acids to various lipids (Fig. 3B). Notably, more abundant metabolites were glycerophosphocholine (GPC), described as an intermediate of lipid remodeling in Saccharomyces cerevisiae, and the glycerophospholipid 1-stearoyl-glycosylphosphatidylinositol (GPI) (46). One of the less abundant intracellular metabolites was farnesol, a well-studied quorum sensing molecule which accumulates extracellularly in dense cultures and inhibits hypha formation (47). Further, cystathionine, a cysteine biosynthesis intermediate, and adenosine as well as their precursor *S*-adenosylhomocysteine were generally present in lower abundance in hyphae. Oxidized glutathione (GSSG) and the two closely linked amino acids citrulline and proline also displayed lowered abundance. Particularly interesting was the presence of three consecutive intermediates from the *de novo* sphingolipid biosynthesis: 3-ketosphinganine, sphinganine, and phytosphingosine (Fig. 3B). The metabolic response in germinating hyphae after 90 min was markedly smaller with only 8 metabolites consistently less abundant (Fig. S5A). Notably, the only metabolite present in both core sets was sphinganine.

10.1128/msystems.00539-22.5 FIG S5 Metabolic response after 90-min incubation in hypha-inducing media in the WT and filamentation-impaired strains and KEGG pathway enrichment analysis for *cph1*Δ *efg1*Δ strain. Metabolites significantly affected in abundance (*P* < 0.05) under the indicated conditions. More abundant metabolites are indicated in blue, and less abundant are indicated in red. (A) Metabolites affected in the WT by the yeast-to-hypha transition (HS and GlcNAc versus SD, pH 7.4 versus pH 4) after 90 min. Abundance changes (log_2_) of the respective core set metabolites (underlined in the Venn diagram) are displayed. (B) Metabolites with different abundances in *hgc1*Δ and *cph1*Δ *efg1*Δ strains compared to the WT after 90-min incubation in the indicated medium. (C) Metabolites significantly affected in abundance in *cph1*Δ *efg1*Δ strain versus WT in the indicated medium (GlcNAc, pH 7.4, and HS) at 240 min were used to identify enriched KEGG pathways via “MetaboAnalyst” enrichment analysis. Only metabolites with a unique HMDB identifier were used. The analysis was performed twice for each comparison with more abundant (blue) or less abundant (red) metabolites. Blue bars indicate potentially less active pathways, and red bars indicate more active pathways. Enriched pathways are indicated for two cutoff values: *P* < 0.05 (dark colors), and 0.05 < *P* < 0.1 (light colors). Dashed lines indicate the canonical significance threshold, *P* = −log_10_(0.05). Download FIG S5, TIF file, 0.9 MB. Copyright © 2022 Garbe et al. 2022 Garbe et al. https://creativecommons.org/licenses/by/4.0/ This content is distributed under the terms of the Creative Commons Attribution 4.0 International license.

When we compared the metabolite changes upon hypha induction after 90 and 240 min, we noted that in HS and pH 7.4 a substantial number of metabolites displayed consistent changes at both time points (Fig. 3B and Fig. S5A). In addition, a comparable number of metabolites was affected at only one of the time points, indicating time-dependent effects. Few changes were visible after 90 min of incubation in GlcNAc, though this markedly increased at 240 min, pointing to slower adaptation (Fig. 3B and Fig. S5A). This further reflects the slower hypha formation than that by the other two stimuli (Fig. S1A).

**(iii) Hyphae are metabolically distinct from yeast.** Next, we conducted an enrichment analysis for KEGG pathways using MetaboAnalyst to identify potentially affected metabolic pathways during filamentation. Previously, metabolite accumulation was described as an indicator of lowered pathway activity and reduced metabolic flux and vice versa (48). The enrichment analysis was performed with both significantly more and significantly less abundant metabolites for each medium condition to identify pathways with potentially lowered or increased activity.

**FIG 4:**
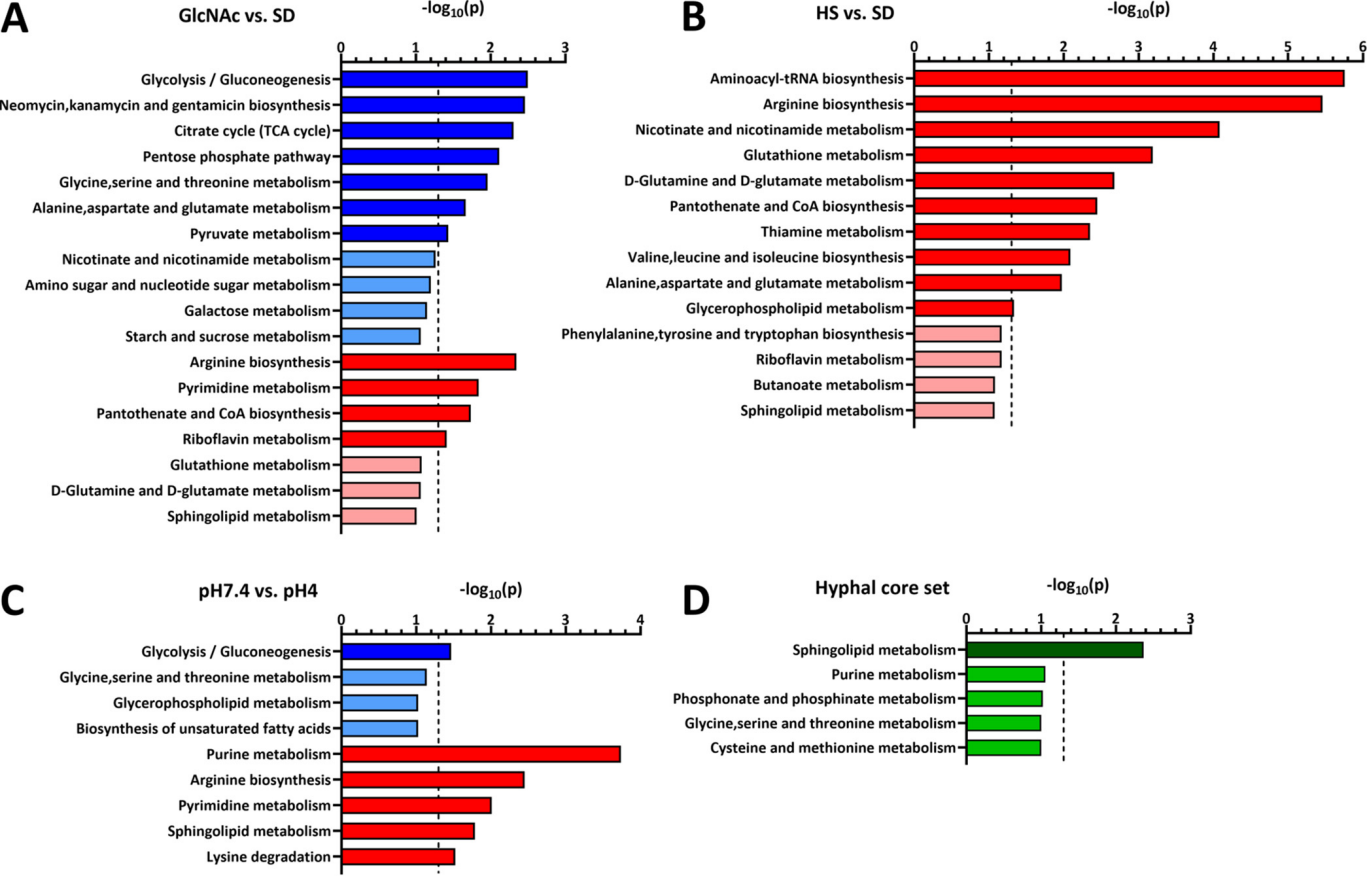
KEGG pathway enrichment analysis for metabolites altered in abundance during WT hypha formation. Metabolites significantly affected in abundance (*P* < 0.05) under the indicated conditions (WT, 240 min) (GlcNAc versus SD [A], HS versus SD [B], pH 7.4 versus pH 4 [C], and metabolic core set [D]) with a unique HMDB identifier were used to identify enriched KEGG pathways via “MetaboAnalyst” enrichment analysis. The analysis was performed twice for each comparison with more abundant (blue) or less abundant (red) metabolites or both (green) for the core set. Blue bars indicate potentially less active pathways, and red bars indicate more active pathways. Enriched pathways are indicated for two cutoff values: *P* < 0.05 (dark colors) and 0.05 < *P* < 0.1 (light colors). Dashed lines indicate the canonical significance threshold [*P* = −log_10_(0.05)].

In HS no pathways were enriched among the more abundant metabolites, while in pH 7.4 only glycolysis/gluconeogenesis reached the significance threshold (Fig. 4B and C). In GlcNAc, however, aside from this pathway others were also significantly enriched, like the tricarboxylic acid (TCA) cycle or pentose phosphate pathway—potentially reflecting a reduced activity of the central carbon metabolism (Fig. 4A). Interestingly, under all three conditions arginine biosynthesis was significantly enriched in the sets of less abundant metabolites, likely indicating a conserved activation of this pathway upon hypha formation. Further, nucleotide-related pathways like pyrimidine (GlcNAc and pH 7.4) or purine (pH 7.4) metabolism were also enriched. The same holds true for pathways associated with vitamin and cofactor metabolism, like pantothenate and coenzyme A (CoA) biosynthesis (GlcNAc and HS) or thiamine metabolism (HS).

For the core set of metabolites with consistently increased or lowered abundance during the hyphal switch, only sphingolipid metabolism met the canonical significance threshold of 0.05 (Fig. 4D). Notably, in the stimulus-specific comparisons, sphingolipid metabolism was significantly enriched for less abundant metabolites in pH 7.4 (Fig. 4B), while it barely missed the cutoff (*P* < 0.05) in GlcNAc (Fig. 4A) and HS (Fig. 4C), suggesting a general trend toward increased activity of this pathway during hypha formation. Although we did not find evidence for a general downregulation of the central carbon metabolism in hyphae as previously stated by Han et al. (35), our results illustrate specific metabolic differences between yeast and hyphae and signature metabolic rearrangements during filamentation.

**FIG 5:**
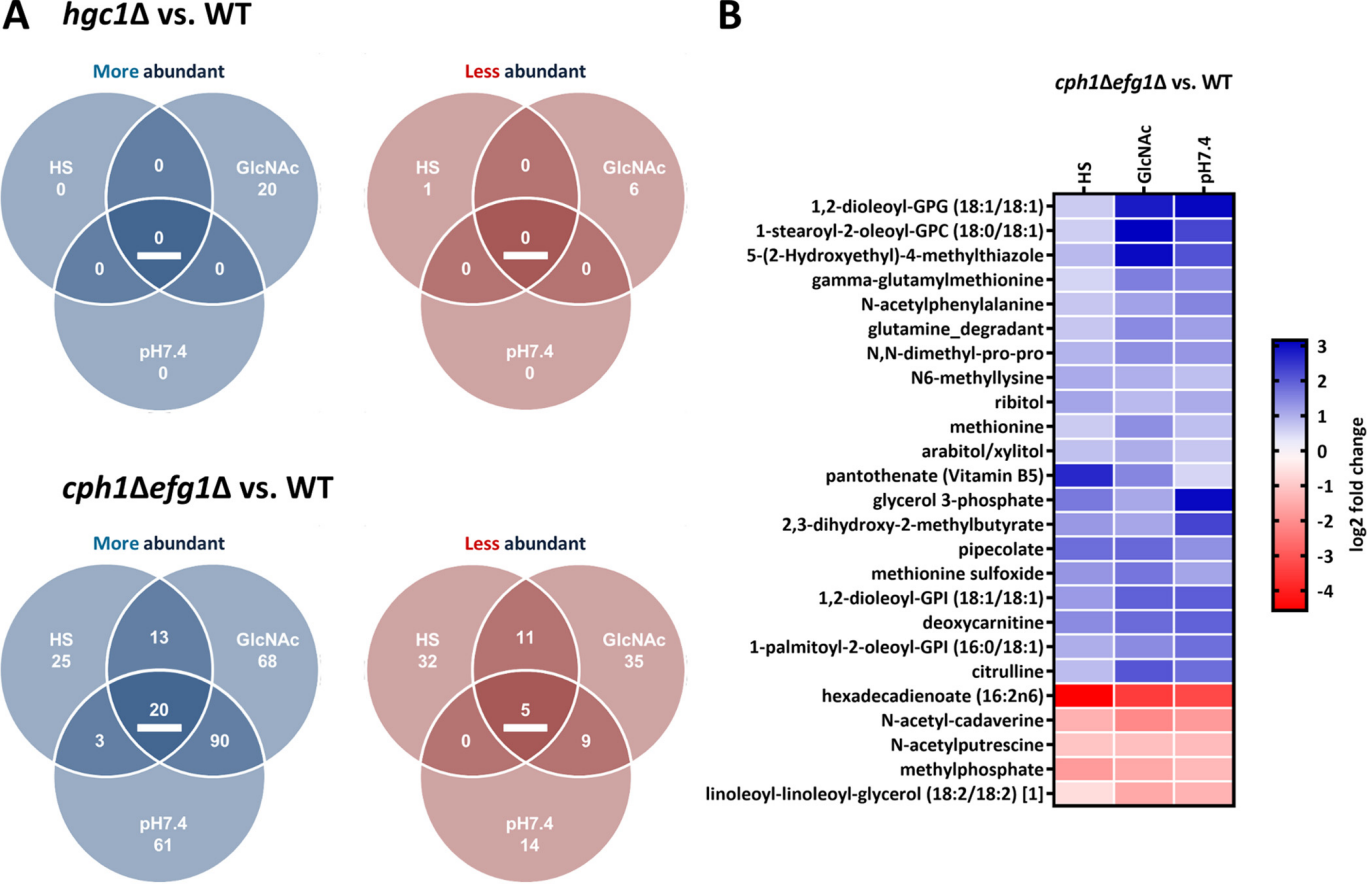
Differentially abundant metabolites in filamentation-impaired mutants in hypha-inducing media. (A) Metabolites significantly affected in abundance (*P* < 0.05) in *hgc1*Δ strain versus WT and *cph1*Δ *efg1*Δ strain versus WT after 240-min incubation in the respective medium (HS, GlcNAc, and pH 7.4). More abundant metabolites are indicated in blue, and less abundant are in red. (B) Abundance changes (log_2_) of the *cph1*Δ *efg1*Δ strain versus WT core set metabolites under the indicated conditions at 240 min.

**(iv) The *hgc1*Δ strain has a WT-like metabolome, while the *cph1*Δ *efg1*Δ strain is metabolically distinct.** Lastly, we investigated potential connections between the metabolic profile of a cell and its respective morphotype. As was already apparent in the previous analysis (Fig. 1B and Fig. S4), the *hgc1*Δ strain is similar to the WT in its metabolic composition despite its vastly restricted ability to filament at 240 min (Fig. 5A and Fig. S1A). Metabolic rearrangements in response to hypha-inducing media appear uncoupled from actual filamentation, analogous to hypha-associated gene expression in the *hgc1*Δ strain, which is also uncoupled from filamentation. However, two notable exceptions were observed for the *hgc1*Δ strain versus the WT—192 metabolites at 90 min in pH 7.4 and 26 metabolites at 240 min in GlcNAc were significantly altered in abundance (Fig. 5A and Fig. S5B). While the differences in pH 7.4 completely vanished at 240 min, the differences in GlcNAc occurred only after 240 min, which peculiarly correlates with the retained capacity of the *hgc1*Δ strain for filamentation in this medium.

The comparison of the *cph1*Δ *efg1*Δ strain with the WT revealed broader changes in the metabolite abundance between the two strains in hypha-inducing media (Fig. 5A and Fig. S5B). Furthermore, we identified a core set of 25 metabolites with consistently different abundance (Fig. 5B). Notably, compared to the WT, the *cph1*Δ *efg1*Δ strain displayed a tendency to metabolite accumulation rather than depletion. In accordance with these findings, we found several pathways enriched in the metabolite sets with higher abundance in the *cph1*Δ *efg1*Δ strain than in the WT, like arginine biosynthesis (GlcNAc and pH 7.4) and purine (pH 7.4) and thiamine (GlcNAc and HS) metabolism (Fig. S5C). As these findings were the inverse of the observed WT-associated changes, this points to a filamentation-associated activation of specific pathways absent in the yeast-locked strain. Notably, sphingolipid biosynthesis barely missed the significance threshold for more abundant metabolites in GlcNAc, while it was significantly enriched in the set of depleted metabolites in HS (Fig. S5C). In summary, the *hgc1*Δ strain maintained a WT-like metabolic fingerprint in hypha-inducing media, while the *cph1*Δ *efg1*Δ strain showed alterations often opposite to the WT-associated changes.

### Sphingolipid biosynthesis is required for C. albicans filamentation.

Most striking in our analysis of the WT metabolic hyphal core set was the specific enrichment of “Sphingolipid metabolism,” namely, by the detection of the initial three consecutive intermediates of the *de novo* sphingolipid synthesis: 3-ketosphinganine, sphinganine, and phytosphingosine. Other intermediates from the biosynthetic pathway were identified as well, displaying a trend toward lowered abundance, although they did not meet our cutoff criteria under all conditions (see “Metabolomics results” at https://doi.org/10.5281/zenodo.7019410). Following the concept that the depletion of intermediates indicates increased pathway activity in hyphae, we hypothesized a connection to filamentation as has been previously shown for filamentous fungi and suggested for C. albicans (48–50).

**FIG 6:**
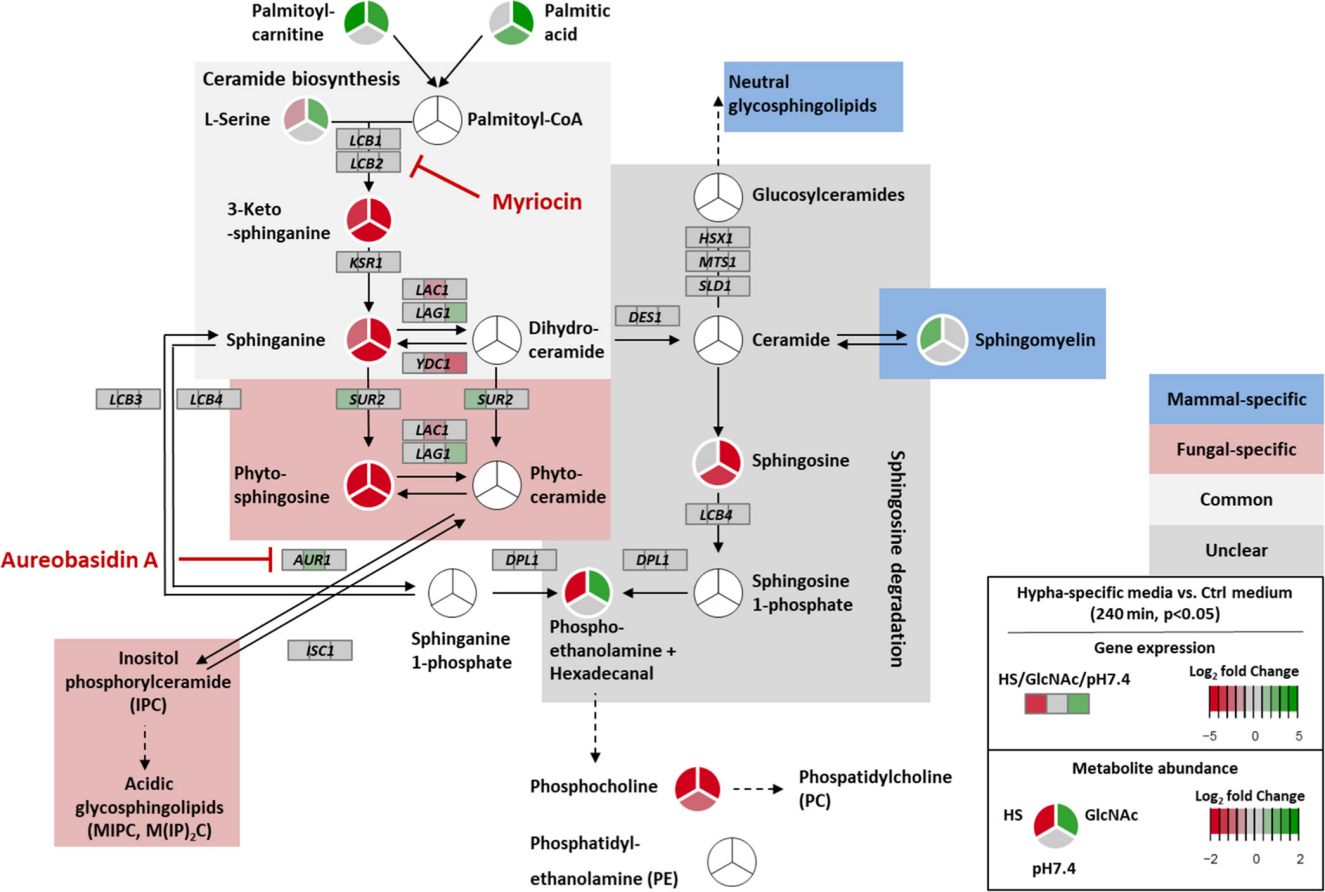
C. albicans sphingolipid biosynthesis is activated during filamentation. Combined projection of transcriptional and metabolic changes of the sphingolipid biosynthesis pathway, indicating depletion of the consecutive initial intermediates—3-ketosphinganine, sphinganine, and phytosphingosine—during WT filamentation, caused by increased pathway activity for the synthesis of required sphingolipids. Circles (for metabolites) or rectangles (for gene expression) are split, displaying the three hyphal conditions (GlcNAc and HS versus SD, pH 7.4 versus pH 4) at 240 min. Green shades indicate gene induction/increased metabolite abundance, and red shades indicate gene repression/lowered metabolite abundance. Changes not meeting the significance threshold (*P* < 0.05) are shown in gray. White circles represent metabolites not detected in our study. Target points of the inhibitors myriocin and aureobasidin A are indicated.

First, we refined the proposed sphingolipid biosynthesis in C. albicans, based on the respective KEGG pathway (cal00600) and current literature for C. albicans and S. cerevisiae (51–57). In this overview we integrated the log_2_ fold changes for the respective genes and metabolites from our transcriptional and metabolic data sets for the 240-min time point (Fig. 6). While the depletion of the first three intermediates was clearly visible, we noticed little to no consistent regulation of the associated genes under all three hypha-inducing conditions. Notably, the lowered abundance of those intermediates could be also observed for 90 min including the GlcNAc-derived samples, where the fungus displayed only germ tube formation and no prominent filamentation (Fig. S1A and “Metabolomics results” at https://doi.org/10.5281/zenodo.7019410). While in the *hgc1*Δ strain almost no differences were apparent, the *cph1*Δ *efg1*Δ strain partially showed the inverse pattern, compared to the WT (“Transcriptomics results” and “Metabolomics results” at https://doi.org/10.5281/zenodo.7019410). Taken together, we concluded a specific and general upregulation of the *de novo* sphingolipid synthesis during the yeast-to-hypha transition.

Based on this, we hypothesized that the inhibition of the pathway would reduce filamentation of C. albicans under hypha-inducing conditions. One possible inhibitor is myriocin (Myr), which is well described and targets the serine palmitoyltransferase that catalyzes the first step of the *de novo* sphingolipid synthesis (Fig. 6) (58). Since myriocin exhibits potent antifungal activity, we first defined a sublethal concentration of the drug that does not interfere with fungal growth and decided for 0.25 μM for all subsequent experiments (Fig. S6A).

10.1128/msystems.00539-22.6 FIG S6 Influence of myriocin and aureobasidin A on C. albicans growth and physiology. (A and B) To determine sublethal concentrations of sphingolipid biosynthesis inhibitors, growth curves in SD and HS were performed in a 96-well microplate reader with the indicated concentrations of myriocin (Myr) (A) and aureobasidin A (AbA) (B). OD_600_ was measured every 20 min. Experiments were performed in biological and technical triplicates. (C) A C. albicans strain carrying GFP under the control of the *ECE1* promoter (*ECE1*p-GFP) was incubated for 240 min in yeast- and hypha-promoting media, in either the absence or the presence of myriocin (Myr). Present hypha-associated gene expression is indicated by a green GFP signal. Bar, 50 μm. (D) C. albicans WT, *hgc1*Δ, and *cph1*Δ *efg1*Δ cells were spotted on the indicated solid hypha-inducing media in either the absence or the presence of phytosphingosine (Phy). Download FIG S6, TIF file, 2.2 MB. Copyright © 2022 Garbe et al. 2022 Garbe et al. https://creativecommons.org/licenses/by/4.0/ This content is distributed under the terms of the Creative Commons Attribution 4.0 International license.

**FIG 7:**
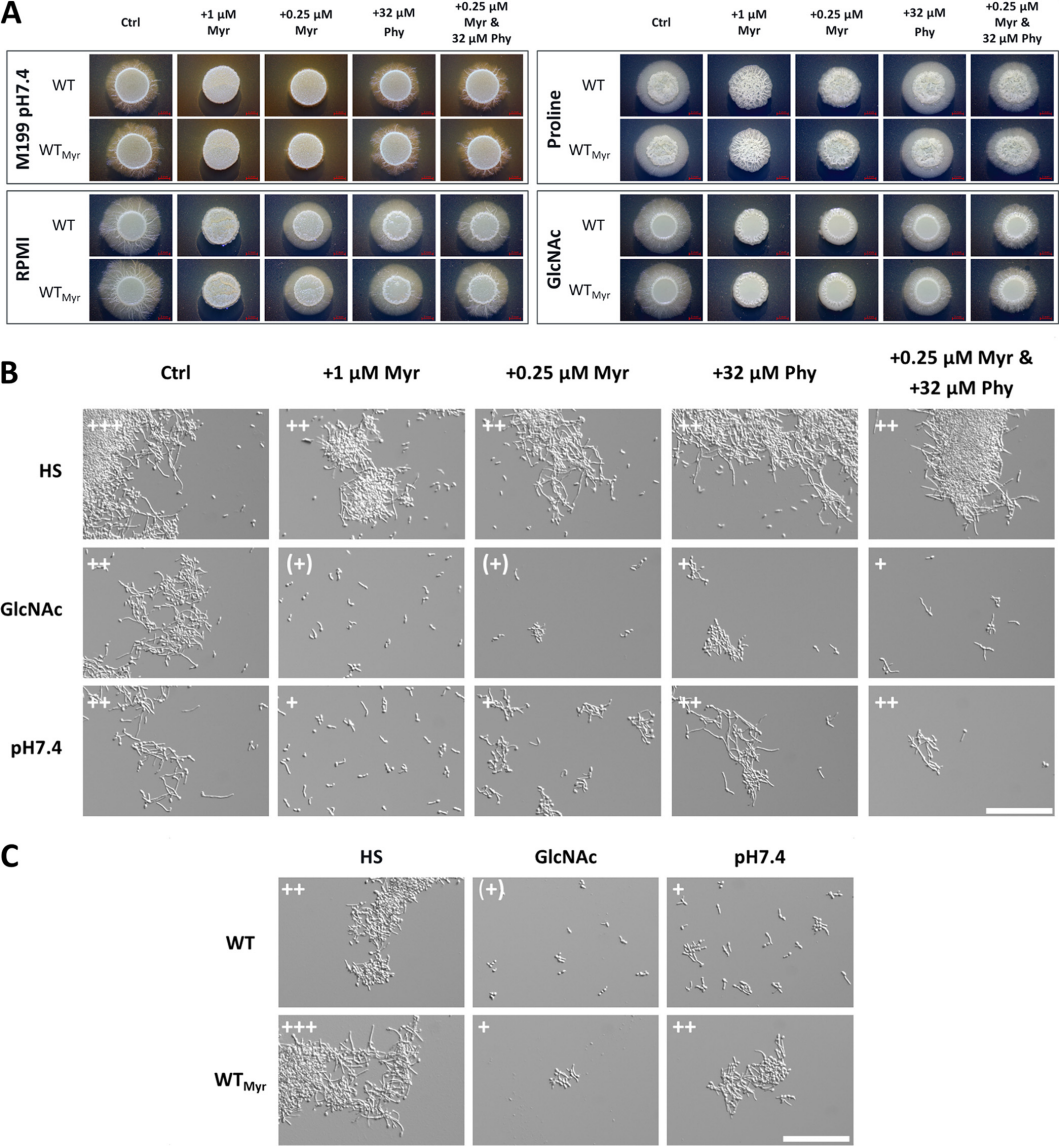
*De novo* sphingolipid biosynthesis is required for C. albicans filamentation. (A) C. albicans WT filamentation is inhibited by myriocin (Myr) on solid, hypha-inducing medium and partially restored by additional supplementation of phytosphingosine (Phy). SD log-phase cells were spotted on the solid medium and incubated in either the absence (WT) or the presence of 0.25 μM myriocin (WT_Myr_) in the preculture. Notably, solidified HS did not induce filamentation and was therefore excluded. (B) C. albicans WT filamentation in liquid, hypha-inducing medium with the indicated supplementation of Myr and Phy after 240 min. (C) SD log-phase cells of the WT, incubated in either the absence (WT) or the presence of 0.25 μM myriocin (WT_Myr_) in the preculture, were transferred to liquid, hypha-inducing medium. Examination of the filamentation after 90 min revealed a hyperfilamentous phenotype for myriocin-pretreated cells (WT_Myr_). Filamentation score: 0, yeast; +, germ tubes and small hyphae; ++, moderate filamentation; +++, extensive filamentation; +, isolated germ tubes. Bar, 100 μm.

Next, we examined the filamentation of C. albicans in response to different hypha-inducing conditions in the presence of Myr on solid media and noted a nearly complete absence of filamentation for all tested conditions (Fig. 7A). Notably, the impaired filamentation was restored by the addition of the downstream intermediate phytosphingosine, while the sole addition of phytosphingosine did not substantially affect the filamentation behavior (Fig. 7A). A slight reduction in the size of the hyphal corona was visible (e.g., RPMI and GlcNAc), which indicates that high levels of these intermediates also affect filamentation. Similar results were obtained in liquid medium for GlcNAc and pH 7.4, while serum-mediated hypha induction represented a notable exception where unaltered filamentation was visible in the presence of Myr (Fig. 7B). A possible explanation is that human serum contains a great variety of sphingomyelins and sphingosine, whose utilization and intracellular conversion to other sphingolipids by yet-undefined mechanisms could circumvent the myriocin effect (Fig. S4B and “Metabolomics results” at https://doi.org/10.5281/zenodo.7019410). Accordingly, C. albicans showed unaffected growth in HS even in the presence of elevated Myr concentrations (Fig. S6A). Further, we observed that C. albicans yeast cells pretreated with Myr, by addition to the SD log-phase preculture, showed a hyperfilamentous phenotype upon the transition to hypha-inducing conditions, specifically prominent at earlier filamentation stages (Fig. 7C). This observation was made only in liquid and not on solid medium, thus likely indicating a transient effect (Fig. 7A and C).

To verify if myriocin-treated cells still possess hypha-associated gene expression despite the impairment in filamentation as previously suggested (37), we utilized a strain carrying a green fluorescent protein (GFP) reporter under the control of the *ECE1* promoter (59). Since this strain showed a GFP signal in hypha-inducing medium with Myr despite impaired filamentation, we concluded that the inhibition of the sphingolipid biosynthesis does not interfere with hypha-associated gene expression (Fig. S6C). Lastly, we tested if the addition of phytosphingosine to hypha-inducing media could restore hyphal growth in the nonfilamentous *hgc1*Δ and *cph1*Δ *efg1*Δ strains. No visible hypha formation was observed for either strain on the tested media (Fig. S6D). Altogether, our data strongly imply that the increased *de novo* synthesis of sphingolipids is pivotal for C. albicans filamentation and that the activation of the pathway generally correlates with hypha formation.

**FIG 8:**
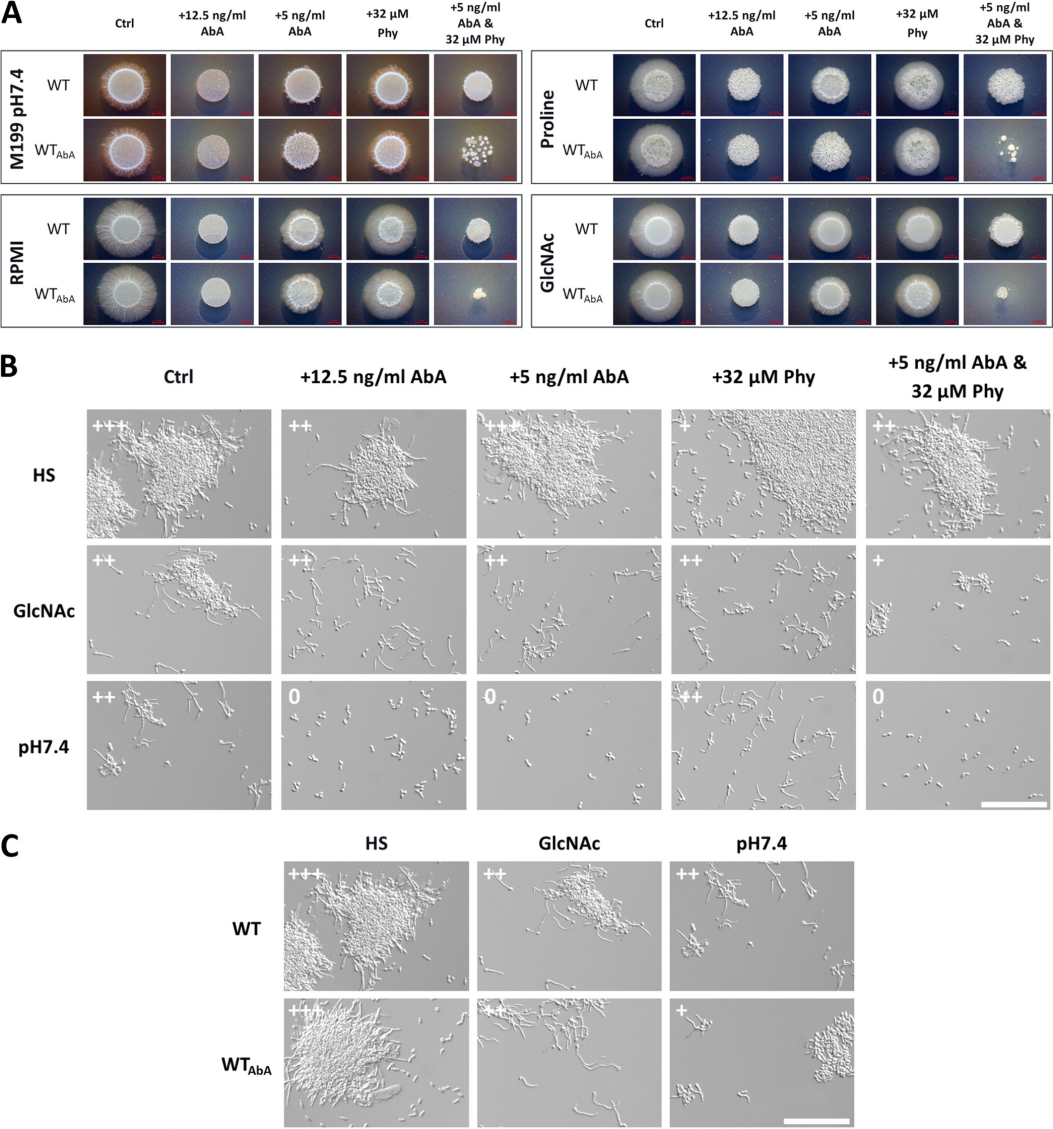
The acidic GSL synthesis inhibitor aureobasidin A blocks C. albicans filamentation in a medium-dependent way. (A) The respective solid, hypha-inducing media were supplemented with the indicated concentrations of aureobasidin A (AbA) or phytosphingosine (Phy). WT SD log-phase cells were spotted on the solid medium and were incubated in either the absence (WT) or the presence of 5 ng/mL AbA (WT_AbA_) in the preculture. Combined supplementation with AbA and Phy resulted in increased growth inhibition, especially for AbA-pretreated cells (WT_AbA_). (B) C. albicans WT filamentation in liquid, hypha-inducing medium with the indicated supplementation of AbA and Phy after 240 min. (C) SD log-phase cells of the WT, incubated in either the absence (WT) or the presence of 5 ng/mL AbA (WT_AbA_) in the preculture, were transferred to liquid, hypha-inducing medium. Filamentation was examined after 240 min, and pretreated cells displayed no hyperfilamentous phenotype. Filamentation score: 0, yeast; +, germ tubes and small hyphae; ++, moderate filamentation; +++, extensive filamentation. Bar, 100 μm.

Since the addition of phytosphingosine, a precursor for the synthesis of acidic GSLs, restored hyphal growth in myriocin-treated cells, we hypothesized that this particular fungus-specific branch of the sphingolipid synthesis is necessary for filamentation. Thus, analogously to the myriocin approach, we utilized aureobasidin A (AbA), which targets the fungus-specific inositolphosphorylceramide (IPC) synthase, therefore inhibiting the acidic GSL synthesis (Fig. 6) (60–62). Since aureobasidin A is a highly effective antifungal, we again determined a sublethal concentration that does not affect fungal growth (Fig. S6B). As expected, the addition of this drug led to the inhibition or strong reduction of filamentation on all tested solid media (Fig. 8A). The additional supplementation with phytosphingosine did not restore filamentation and instead led to increased growth inhibition, especially in cells pretreated with AbA, while it did not affect pretreated cells when applied alone. This observation again pointed to potential detrimental effects of elevated phytosphingosine concentrations. In liquid medium the filamentation in the presence of AbA was unaltered in HS and, in contrast to myriocin treatment, also in GlcNAc but still absent in pH 7.4 (Fig. 8B). A hyperfilamentous phenotype for aureobasidin A-pretreated cells shifted to hypha-inducing conditions was not observed (Fig. 8C). In fact, in pH 7.4 the filamentation even appeared to be reduced. Taken together, these findings illustrate the particular importance of acidic GSL synthesis upon yeast-to-hypha transition and the necessity for a tight balance of the respective precursors, like phytosphingosine.

## DISCUSSION

The polymorphism of C. albicans, especially the yeast-to-hypha transition, is considered to be its key virulence factor and consequently is extensively studied. While morphotype-associated gene expression and underlying regulatory circuits have been extensively investigated, accompanying metabolic changes have so far remained understudied. Recent work by Han et al. identified major changes in the metabolite abundances of cells undergoing hypha transition and hypothesized a global downregulation of the central carbon metabolism (35). However, it is noteworthy that compared to recent metabolome analyses, a limited number of metabolites was detected in this study and metabolite classes like lipids were underrepresented (36, 37). To expand those previous results, here we combined transcriptional and metabolic profiling approaches to investigate the fungal metabolic response to filamentation on two levels, gene regulation and metabolite changes.

Previously, Martin et al. first described core filamentation response genes—a group of eight genes whose expression is associated with hyphal growth and independent of the applied stimulus (21). This set was verified, except for *HGT2*, and further extended in our study to 33 genes with consistent up- or downregulation in germinating and elongated hyphae. For 240 min, where prominent hypha formation was visible for all three stimuli, we identified 82 genes with consistent differential expression. Yet, for these genes we did not find a clear enrichment of metabolic processes except for “carbohydrate transport”—a very general term also containing primarily virulence-associated genes, like *ECE1*, *HWP1*, or *ALS3*. Nevertheless, single genes clearly associated with metabolism were consistently upregulated in elongated hyphae, e.g., those coding for the alternative oxidase AOX2 or the amino acid permeases MUP1 and GAP6, while the genes for the sugar transporters *HGT6*, *HGT8*, and *HXT5* displayed downregulation (63–66).

Further, when comparing the core sets of up- or downregulated genes during filamentation after 90 and 240 min, we noticed significant differences. Those were, at least in part, attributed to the faster return to yeast expression levels in serum-induced hyphae, compared to the induction by alkaline pH or GlcNAc. This might point to specific characteristics of the serum induction, since the filamentation itself increased between 90 and 240 min. We can only speculate about the underlying causes, but one explanation might be that serum is a multifactorial stimulus indicating a hostile host environment, which requires more rapid adaptation by the fungus than do the two other stimuli.

Analogous to the core set of upregulated genes, we identified a comparable number of genes consistently downregulated in elongated hyphae and a smaller set during germination. Applying the same logic as for the hypha-associated genes, we propose that further examination of these genes with yeast-associated expression could yield novel insights into the biology of the C. albicans dimorphism.

On the metabolic side, we found the C. albicans metabolomes predominantly clustered by growth medium and time points, suggesting that in contrast to the transcriptome these have a greater impact on the cellular metabolism than the morpho- or genotype. This is also supported by the lack of consistent genotype-independent correlation between morphotype and the metabolome. While the *hgc1*Δ strain displayed a mainly WT-like metabolome, the *cph1*Δ *efg1*Δ strain markedly differed from the WT, for instance in the metabolite abundance for pathways like arginine biosynthesis or nucleotide and vitamin metabolism. Whether this is linked to the absent filamentation or attributed to other regulatory functions of the two deleted transcription factors, which were previously shown to be involved in metabolic regulation, requires further investigation (67–70).

In WT hyphae we found a variety of pathways enriched in the metabolite sets with differential abundance, yet no evidence for a general downregulation of the central carbon metabolism as previously proposed (35, 48). In fact, we rather observed morpho- and medium-dependent metabolic rearrangement processes, like potentially increased arginine biosynthesis. Interestingly, the specific induction of arginine biosynthesis was also shown in C. albicans cells confronted with macrophages, which also trigger hyphal growth (71). Remarkably, the sole pathway enriched in the stimulus-independent metabolic core set was the sphingolipid metabolism.

Sphingolipids, especially GSLs, fulfill a variety of biological functions in the cell and are involved in cell polarity, morphogenesis, signaling, and stress adaptation (49, 50, 72–74). They play a key structural role for cellular membranes as they are often enriched in functional microdomains (75). In fungi two main classes are present—neutral and acidic GSLs. More prominent in plants and mammals, the variety of neutral GSLs in fungi is smaller by far with glucosylceramide and galactosylceramide as the considered endpoints. Acidic GSLs, on the other hand, account for the largest proportion of GSLs in yeast and represent a much more diverse group, whose synthesis is in part fungus specific, making them promising drug targets (56, 57, 76). Despite their biological importance, sphingolipids and their synthesis have not been extensively or systematically studied in C. albicans (50). Nevertheless, previous studies already characterized parts of their synthesis and physiological relevance and linked sphingolipids to cell polarity and filamentous growth under certain conditions (77–82).

Here, we identified the activation of *de novo* sphingolipid biosynthesis as hypha associated and essential for C. albicans filamentation, independent of the hypha-inducing stimulus. The addition of sublethal concentrations of myriocin, a well-studied inhibitor of the initial step of the sphingolipid biosynthesis, consequently led to impaired or absent hyphal growth, which is in accordance with a previous study (82). In contrast to the work of Martin and Konopka, who used significantly higher concentrations of myriocin, we did not observe inhibition of serum-induced filamentation (82). Thus, we hypothesize that the fungus is able to utilize serum components, namely, sphingomyelins, to compensate for the impaired *de novo* synthesis. The increased variety of ceramides absent in human serum but specifically detected in fungal cells obtained from serum samples supports this assumption. Still, it remains unclear if sphingomyelins are directly taken up via transporters or endocytosis or if membrane-bound sphingomyelinases extracellularly generate ceramides, which are then utilized by the fungus. The presence of functional sphingomyelinases necessary for both possibilities and described for other fungi like S. cerevisiae has not yet been verified for C. albicans, and it remains an open question as to how the fungus could use these host-derived sphingolipids (83–85).

The myriocin-induced filamentation impairment was reversed by the addition of phytosphingosine, which suggests that the synthesis of acidic GSLs is required for C. albicans filamentation. Phytosphingosine is a precursor for IPC generation, which is subsequently used for the synthesis of acidic GSLs, like glycosylinositol (GIPC) and mannosylinositol and mannosyldiinositol phosphorylceramides [MIPC and M(IP)_2_C] (49), though it is not yet clear if ceramide can be synthesized from exogenous phytosphingosine and used for further synthesis of neutral GSLs, like glucosylceramide, which was recently linked to fungal virulence (78).

Our data further revealed impaired filamentation upon specific targeting of the IPC synthase by aureobasidin A, supporting the assumption that acidic GSLs are particularly relevant for filamentous growth. Previous studies further support this. For instance, the deletion of the 3-ketosphinganine reductase Ksr1 in C. albicans resulted in reduced intracellular IPC levels, increased sensitivity to aureobasidin A, and absent filamentation in response to bovine serum (77). Further, the deletion of ceramide synthase Lag1, which generates precursors for IPC synthesis, also resulted in absent filamentation in response to different stimuli (79). Notably, the deletion mutant of the second ceramide synthase Lac1, which generates precursors for neutral GSL synthesis, was unaffected in filamentation (79). Lastly, C. albicans cells depleted of *IPT1*, which is required for the synthesis of M(IP)_2_C, are affected in hypha formation in response to different stimuli and in adhesion (80, 81).

IPC synthases are reportedly essential for fungal viability and are therefore considered promising drug targets (76, 86). Indeed, we could observe a strong impairment of fungal growth even with low levels of aureobasidin A. In addition to the impaired synthesis of acidic GSLs, the impaired growth is likely caused by the accumulation of IPC precursors like phytosphingosine and ceramides. Accordingly, we observed increased growth inhibition when the IPC precursor phytosphingosine was coadministered with aureobasidin A. Typically, the respective levels are under tight regulation due to their involvement in stress adaptation or apoptosis, which can be caused by ceramide accumulation (87–89).

Throughout our data we observed little to no consistent change in the expression levels of the sphingolipid biosynthetic enzymes, suggesting mainly posttranscriptional regulatory mechanisms. In accordance with this, myriocin treatment was previously shown to strongly affect the phosphoproteome in S. cerevisiae and lead to minor transcriptional changes of sphingolipid biosynthetic genes in C. albicans (37, 90). Further, myriocin treatment did not lead to downregulation of hypha-associated genes, which was also verified in our study (37). Thus far, the mechanisms regulating sphingolipid metabolism in C. albicans are only partially understood. The transcription factors Rtg1 and Rtg3 are involved in maintaining sphingolipid homeostasis and regulation of the respective biosynthetic enzymes (37). In S. cerevisiae the kinase Swe1 was shown to regulate sphingolipid biosynthesis, where the deletion results in strongly reduced intracellular phytosphingosine levels and abnormal cell morphology (91, 92). While the function of the C. albicans ortholog in the regulation of the cell cycle and size was already reported, the investigation of its potential role in the sphingolipid metabolism is pending (93–95).

Interestingly, we further observed that C. albicans yeast cells pregrown in the presence of myriocin displayed a hyperfilamentous phenotype upon transition to hypha-inducing conditions. We hypothesize that the intracellular levels of the initial intermediates play an important role in regulating the respective pathway activity by certain feedback loops. Depletion by myriocin pretreatment might simulate a high demand, “priming” the respective enzymes and finally leading to rapid adaptation and hyperfilamentation upon the onset of hypha induction. Another possibility would be that the inhibition of *de novo* synthesis activates the rheostat, which mediates intracellular conversion of sphingolipids into the particular sphingolipid classes required for the yeast-to-hypha transition (96, 97). Nevertheless, these effects would likely be transient, as supported by the absence of hyperfilamentous growth on solid medium. However, future research like phosphoproteome analyses will be required to decipher the C. albicans regulation of sphingolipid homeostasis and its interconnections with other regulatory pathways.

Within this study we successfully identified a stimulus-independent metabolic core signature of C. albicans hyphae and the sphingolipid biosynthesis as central for proper filamentation. It serves as an excellent example to illustrate the potential of metabolomic investigations, since transcriptional studies have so far overlooked the relevance of this pathway. Future research will be necessary to detail the physiological role of certain sphingolipid species in C. albicans physiology during filamentation, for instance, by formation of structural membrane microdomains (82, 98). Lastly, we are confident that further studies based on the data presented here will further elucidate metabolic specifics of hyphae, ultimately leading to a better understanding of fungal physiology as a whole.

## MATERIALS AND METHODS

### Medium composition and C. albicans cultivation.

If not explicitly stated otherwise, all chemicals and medium components used in this study were purchased from Sigma-Aldrich. C. albicans strains were generally maintained as glycerol stocks and cultured in YPD (1% yeast extract, 2% peptone, 2% glucose, solidified with 2% agar for plates). Overnight cultures were prepared in liquid synthetic dextrose medium (SD; 2% glucose, 0.17% yeast nitrogen base [YNB] without amino acids, 0.5% ammonium sulfate, pH 4.5) at 37°C with 180-rpm shaking and prior to experimental use reinoculated in SD (starting optical density at 600 nm [OD_600_] of 0.3) and incubated until early log phase (OD_600_ = 1). Test media were prepared as follows: HS, SD with 10% (vol/vol) human serum (male, AB negative); GlcNAc, 2% *N*-acetylglucosamine, 0.17% YNB without amino acids, 0.5% ammonium sulfate, pH 4.5. Ready-to-use medium 199 with Earle’s salts and l-Glu (catalog no. M5017) was buffered to either pH 4 or pH 7.4 by addition of a 1/5 volume of 0.1 M citric acid and 0.2 M Na_2_HPO_4_ buffer.

### Strain construction.

Two independent strains with deletion of *HGC1* were constructed according to the *SAT1*-*FLP* method (99). In brief the plasmid contained the PCR-amplified flanking region (480 bp 5′ and 473 bp 3′) of the *HGC1* open reading frame. Two independent knockout strains with identical phenotypes were created. Strain A was used for all analyses indicated in this study. All strains used in this study are listed in Table S2 in the supplemental material (38, 59, 100).

10.1128/msystems.00539-22.8 TABLE S2 Strain list. Download Table S2, XLSX file, 0.01 MB. Copyright © 2022 Garbe et al. 2022 Garbe et al. https://creativecommons.org/licenses/by/4.0/ This content is distributed under the terms of the Creative Commons Attribution 4.0 International license.

### Sampling protocol for global transcriptional and metabolomic profiling.

C. albicans log-phase cells were harvested by centrifugation, washed twice in YNB (0.17% YNB without amino acids, pH 4.5), and finally resuspended in YNB solution and inoculated into 30 mL of the respective test medium at an OD_600_ of 0.3. At the indicated time points (0, 90, and 240 min), cells were collected by centrifugation, snap-frozen in liquid nitrogen, and stored at −80°C until further processing. For transcriptional and metabolic analysis, each test culture was split—1 mL for RNA isolation and 29 mL for metabolite extraction. Cells for metabolite extraction were washed with 1× phosphate-buffered saline (PBS) prior to freezing. For phenotypic evaluation 50 μL cells per time point and medium was inactivated in 150 μL 4% ROTI Histofix and the morphology was assessed via differential inference contrast (DIC) imaging with a Zeiss AxioObserver Z.1 microscope (Zeiss, Germany) and ZEN2.3 (blue edition) used for image capture. The sampling was performed in four biological replicates. All cultures were incubated with 180-rpm shaking and, to avoid temperature shift-dependent effects, generally at 37°C.

### Transcriptional profiling.

**(i) RNA isolation.** Total RNA extracts from frozen cell pellets were prepared as previously reported (101). RNA quality and quantity were assessed via the RNA Nano 6000 assay kit of the Bioanalyzer 2100 system (Agilent Technologies, CA, USA; Nanochip, plant program, RNA integrity number [RIN] of >8) and a Nanodrop 1000 (Thermo Fisher Scientific, MA, USA).

**(ii) RNA sequencing.** RNA sequencing was performed by Novogene Europe (Cambridge, UK) with 150-bp paired ends and a minimum of 10 million reads per sample. A total amount of 0.4 μg RNA per sample was used as input material for the RNA sample preparations. Sequencing libraries were generated using NEBNext Ultra RNA library prep kit for Illumina (New England Biolabs [NEB], USA) following the manufacturer’s recommendations, and index codes were added to attribute sequences to each sample. Briefly, mRNA was purified from total RNA using poly(T) oligonucleotide-attached magnetic beads. Fragmentation was carried out by Covaris sonication. First-strand cDNA was synthesized using random hexamer primers and Moloney murine leukemia virus (M-MuLV) reverse transcriptase (RNase H^−^). Subsequently, second-strand cDNA synthesis was performed using dTTP. The remaining overhangs were converted into blunt ends via exonuclease/polymerase activities. Afterward, the 3′ ends of DNA fragments were adenylated. Next, an NEBNext adaptor with hairpin loop structure was ligated for hybridization. To select cDNA fragments of preferentially 250 to 300 bp in length, the library fragments were purified with the AMPure XP system (Beckman Coulter, Beverly, MA, USA). Then, 3 μL User enzyme (NEB, USA) was used with size-selected, adaptor-ligated cDNA at 37°C for 15 min followed by 5 min at 95°C before PCR. Then, PCR was performed with Phusion high-fidelity DNA polymerase, universal PCR primers, and index (X) primer. At last, PCR products were purified (AMPure XP system), and library quality and quantity were assessed by Qubit and real-time PCR. The clustering of the index-coded samples was performed on a cBot Cluster Generation System using a cBot-HS PE cluster kit (Illumina) according to the manufacturer’s instructions. After cluster generation, the library preparations were sequenced on an Illumina Novaseq 6000 platform, and 150-bp paired-end reads were generated.

**(iii) Bioinformatic analysis.** The raw data quality was confirmed using FastQC (version 0.11.9). The reference genome (*C_albicans*_SC5314_version_A22-s07-m01-r44_chromosomes.fasta) and genome annotations (*C_albicans*_SC5314_version_A22-s07-m01-r44_features.gtf) were downloaded from the *Candida* Genome Database. Bowtie 2 (version 2.3.5.1) was used to map paired-end reads to the reference genome. Mapped reads were assigned to genomic features using the function featureCounts of the R package Rsubread (version 2.2.6). Individual contrasts between sample groups were calculated using R package DESeq2 (version 1.28.1), and *P* values were adjusted according to the Benjamini-Hochberg method.

**(iv) Gene ontology and Venn analysis.** Gene lists of analyzed contrasts were filtered for significant differences (*P* < 0.05 and log_2_ fold change ≥1 or ≤−1) and analyzed for enriched GO terms (biological process) using the gene ontology term finder of the *Candida* Genome Database (102). The obtained GO terms were summarized to reduce redundancy using REVIGO with a dispensability cutoff of 0.7 (103). All Venn analyses were performed using the online tool http://bioinformatics.psb.ugent.be/webtools/Venn/. Graphical visualization of GO and Venn analyses was done using GraphPad Prism 9.4.

### Metabolic profiling.

** (i) Metabolite extraction and measurement.** Untargeted, global metabolic profiling was carried out by Metabolon, Inc. (Morrisville, NC, USA), including metabolite extraction and measurement and raw data processing. In brief, the sample preparation involved protein precipitation and removal with methanol, shaking, and centrifugation. The profiling of the resulting extracts was performed on an accurate mass global metabolomics platform consisting of multiple arms differing by chromatography methods and mass spectrometry ionization modes to achieve broad coverage of compounds, which differed by physiochemical properties such as mass, charge, ionization behavior, and chromatographic separation. The details of this platform were described previously (104, 105). The metabolites were identified in the experimental samples by automated comparison of the ion features to a reference library of chemical standard entries that included retention time, molecular weight (*m/z*), preferred adducts, and in-source fragments as well as associated mass spectrometry (MS) spectra. For quality control the identification was curated by visual inspection using software developed at Metabolon (106, 107).

**(ii) Bioinformatic analysis.** All metabolite raw data were normalized to cellular protein content in terms of raw area counts between samples. Subsequently, each biochemical was normalized to Bradford-measured protein concentration and rescaled to set the median equal to 1. Then, missing values were set to the value of the smallest nonmissing value for each metabolite. Fold changes between indicated sample groups and *P* values were calculated using a two-sided *t* test. *P* values were adjusted according to the Benjamini-Hochberg method.

**(iii) Metabolite enrichment analysis.** Analyzed contrasts were filtered for metabolites with significantly altered abundance (*P* ≤ 0.05). All metabolites possessing an HMDB identifier were subsequently used to identify enriched KEGG pathways using MetaboAnalyst 5.0 (45) (enrichment analysis with standard settings). Graphical visualization of the results was done using GraphPad Prism 9.4.

**(iv) Hierarchical clustering of metabolites.** Z scores of metabolites were hierarchically clustered using Ward’s minimum variance method and a Euclidean distance between Z scores (108).

### Filamentation assays.

**(i) Liquid filamentation assays.** C. albicans cells were incubated as outlined in the sampling protocol. Where indicated the sphingolipid biosynthesis inhibitors myriocin and aureobasidin A (AbMole BioScience, Belgium) and the sphingolipid precursor phytosphingosine (TCI, Germany) were added to log-phase precultures or filamentation cultures. All three chemicals were dissolved in methanol and stored in glass vials. For microscopic examination, cells were inactivated by addition of 4% ROTI Histofix and the morphology was assessed via DIC imaging with a Zeiss AxioObserver Z.1 microscope (Zeiss, Germany). Hypha-associated gene expression using *ECE1*p-GFP was assessed via fluorescence microscopy. ZEN2.3 (blue edition) was used for image capture.

**(ii) Solid filamentation assays.** The indicated media were solidified with 2% highly purified agar (BD, CA, USA) supplemented with myriocin, aureobasidin A, and phytosphingosine if indicated. The following media were used: GlcNAc (0.1% glucose, 0.17% YNB, 0.5% ammonium sulfate, 5 mM GlcNAc, 25 mM potassium phosphate buffer, pH 7) (109); 1% RPMI (catalog no. 31800105; Thermo Fisher, MA, USA); proline (0.2% glucose, 10 mM proline, 0.17% YNB, pH 6, adapted from the work of Silao et al. [30]); M199 (catalog no. M5017) buffered to pH 7.4.

Drug-pretreated or nontreated log-phase *Candida* cells were washed twice with YNB and set to an OD_600_ of 0.4, and 5 μL cell suspension was spotted onto agar plates. The plates were incubated at 37°C for 4 to 5 days, and colony morphology was assessed using a Stemi 305 (Carl Zeiss, Germany).

### Growth assays.

C. albicans overnight (ON) cultures were washed twice and set to an OD_600_ of 1 in sterile distilled water (dH_2_O), and 10 μL was added to 190 μL of the indicated medium to reach a starting OD_600_ of 0.05. All growth measurements were performed in biological and technical triplicates in a 96-well microplate reader (Infinite 200 Pro; Tecan). The OD_600_ was subsequently measured every 30 min (circle filled, 4 × 4, 750 μM) for 48 h at 37°C with 10 s (3 mm) of shaking and 10 s waiting prior to each measurement. Growth was visualized as mean per time point using GraphPad Prism 9.4.

### Data availability.

Processed transcriptional data are supplied as “Transcriptomics results” available at https://doi.org/10.5281/zenodo.7014351, and raw data are accessible at GEO (accession no. GSE202941). All metabolome data are provided as “Metabolomics results” available at https://doi.org/10.5281/zenodo.7014351.
