# Supplementary figures and images for: Systematic Metabolic Profiling Identifies De Novo Sphingolipid Synthesis as Hypha Associated and Essential for Candida albicans Filamentation
Source: mSystems. 2022 Oct 20;7(6):e00539-22. doi: 10.1128/msystems.00539-22 (PMC9765226; doi:10.1128/msystems.00539-22)

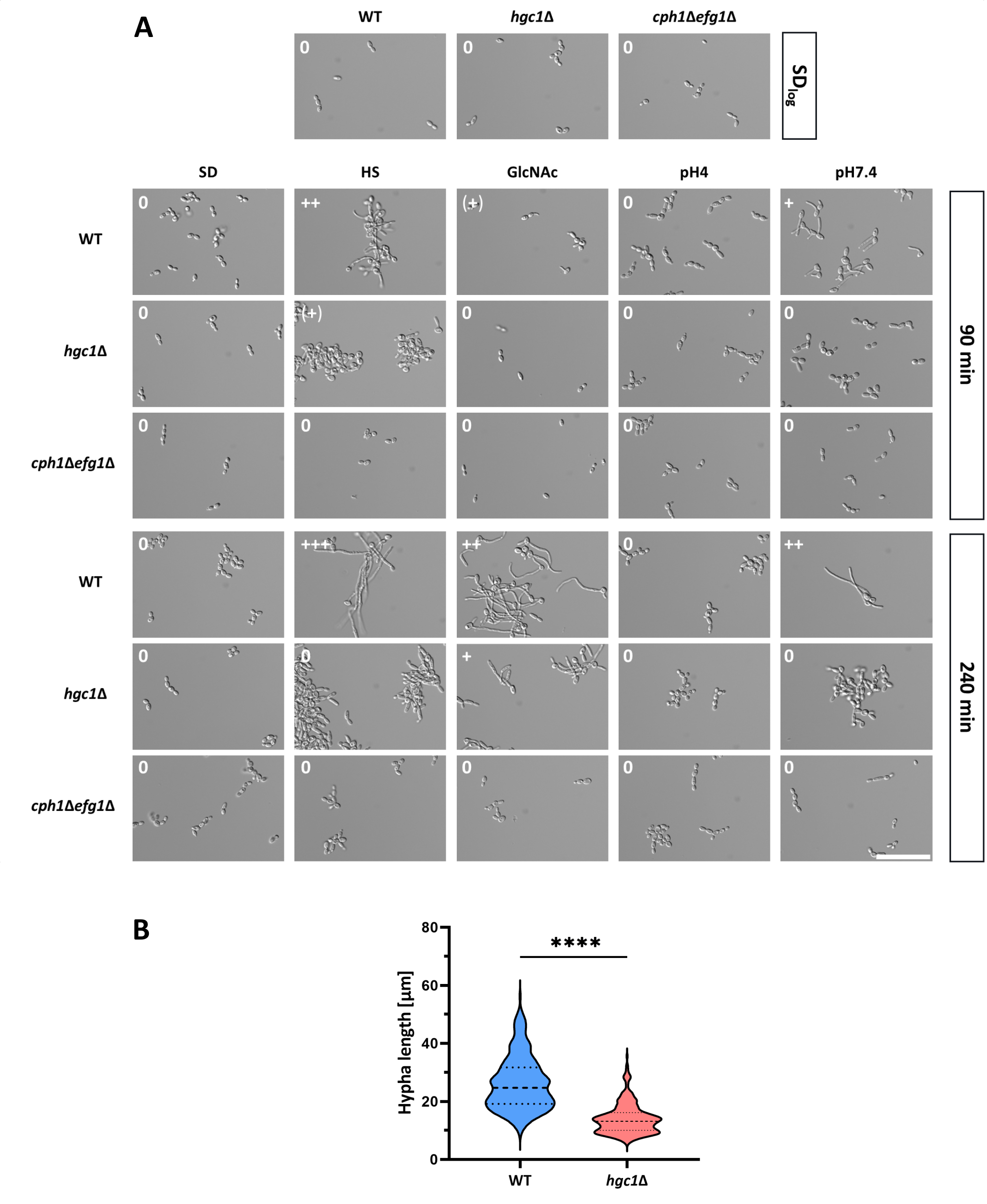

Supplement: FIG S1 [file msystems.00539-22-s0001.tif]

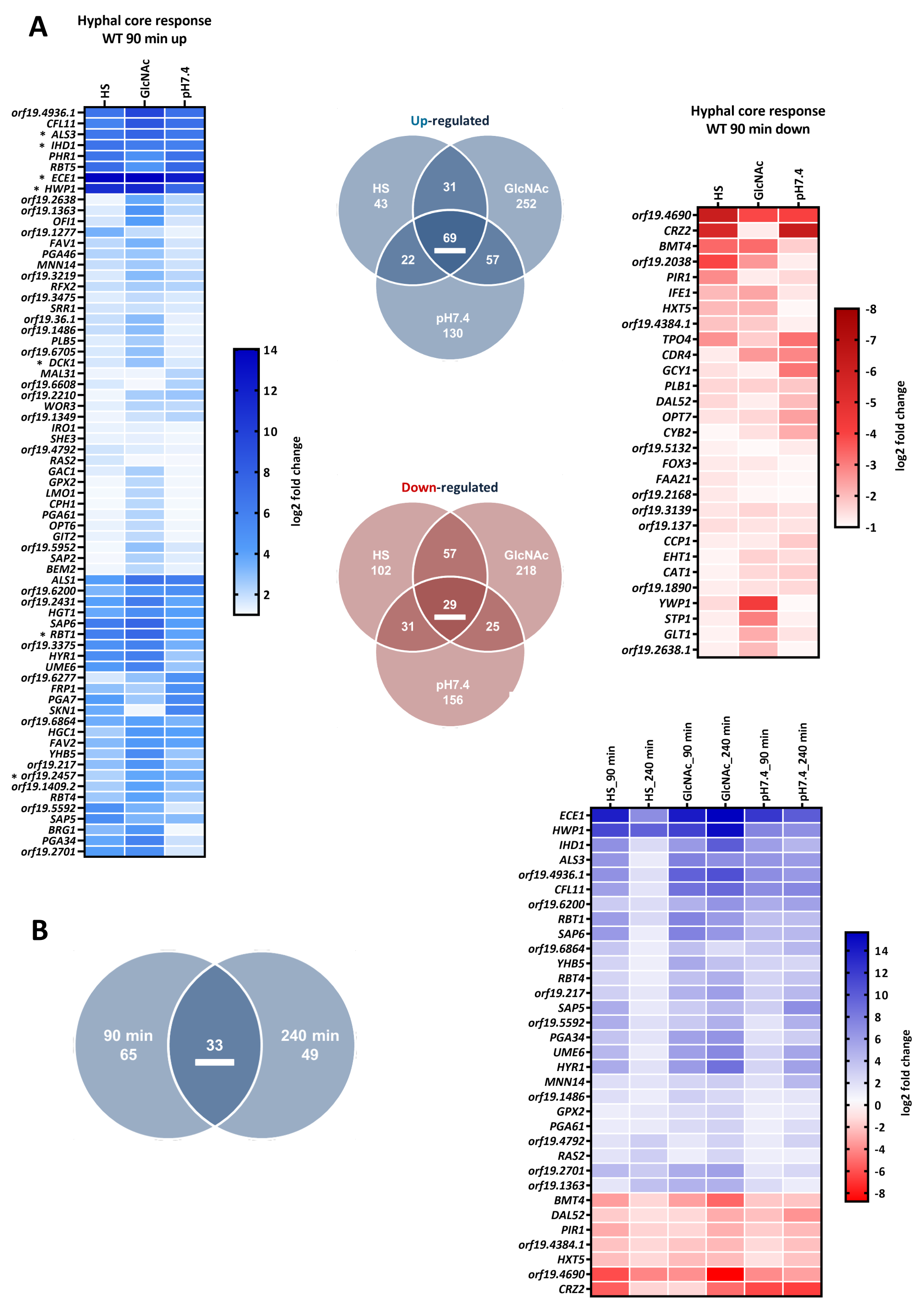

Supplement: FIG S2 [file msystems.00539-22-s0002.tif]

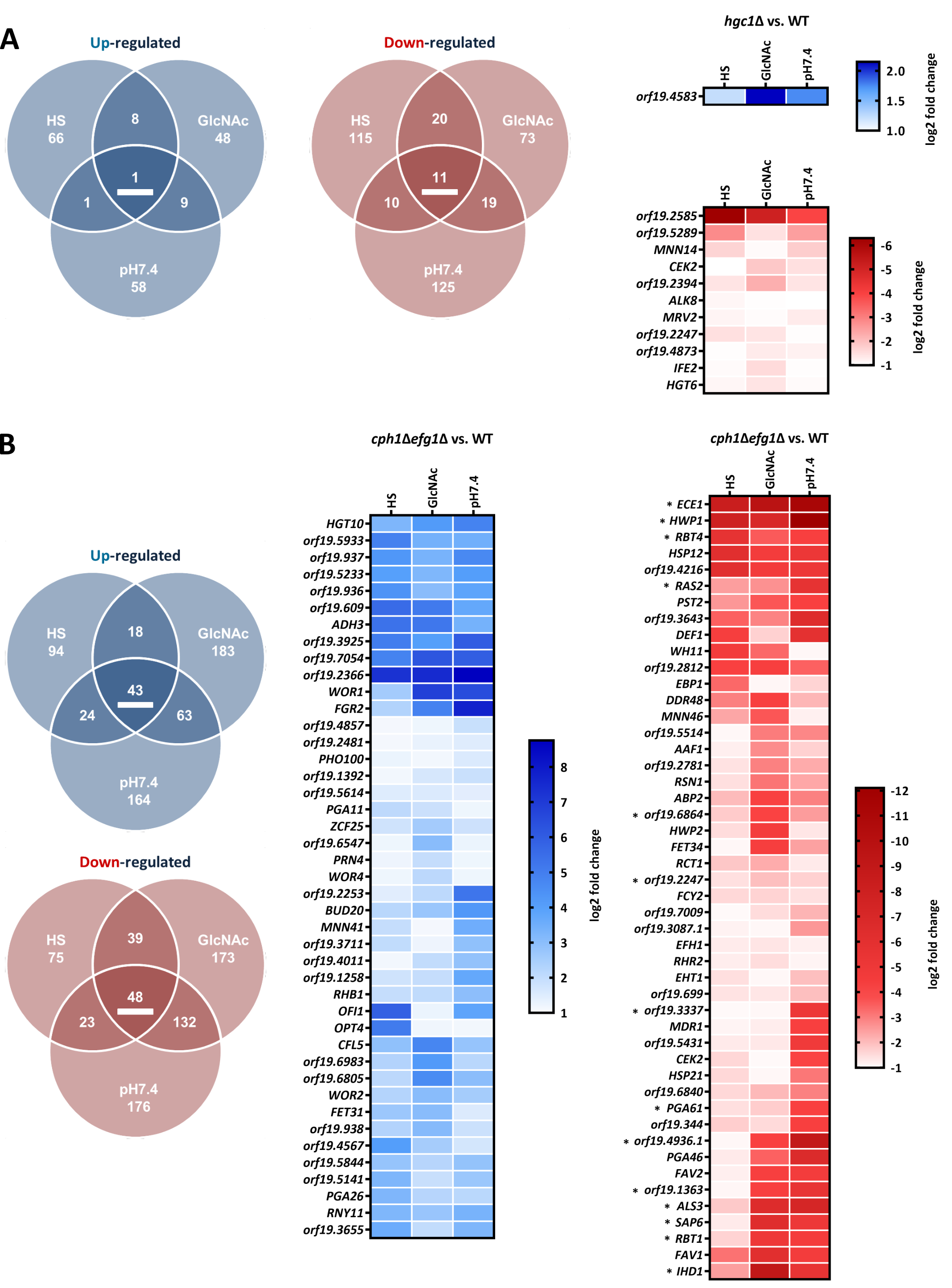

Supplement: FIG S3 [file msystems.00539-22-s0003.tif]

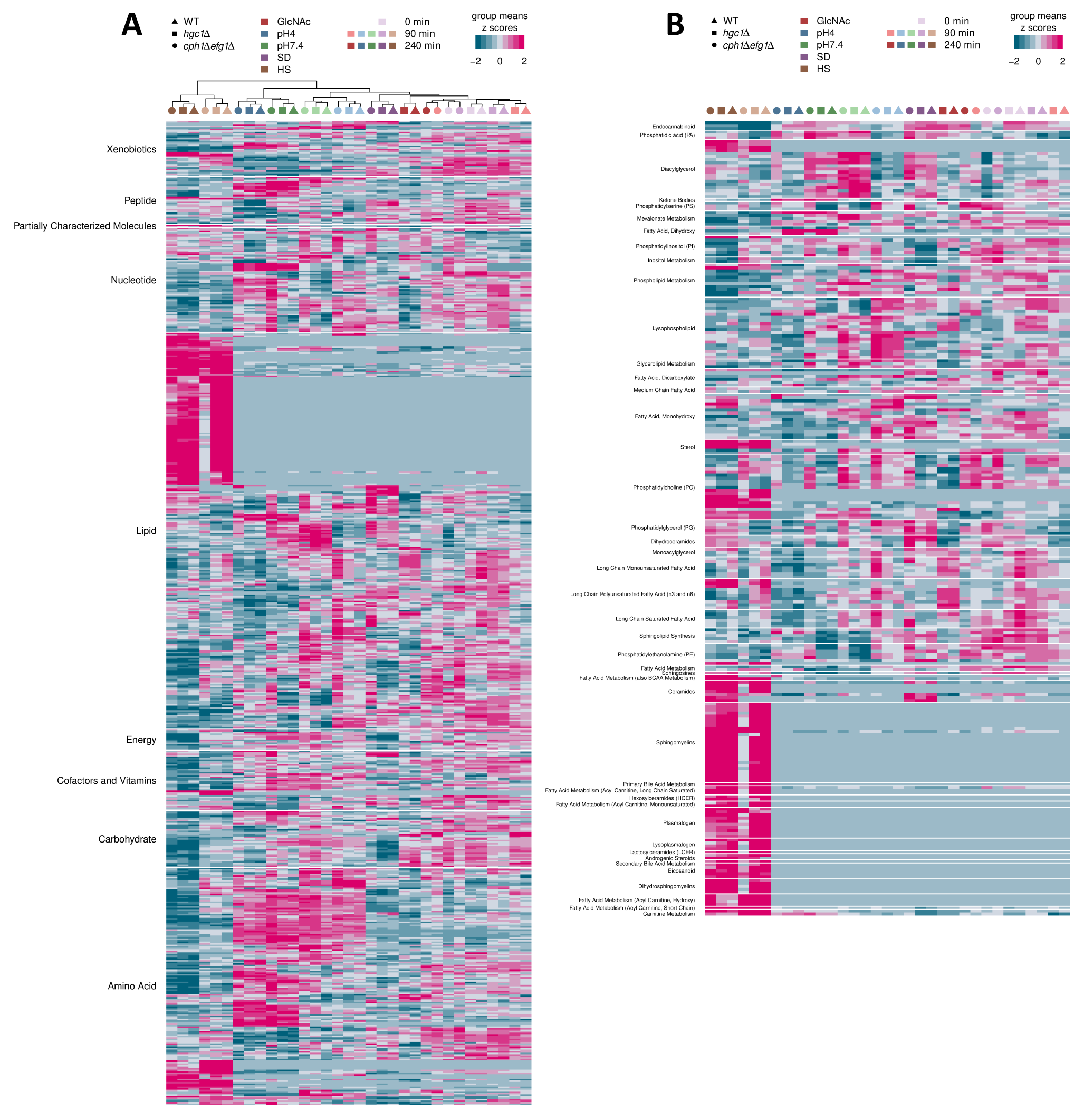

Supplement: FIG S4 [file msystems.00539-22-s0004.tif]

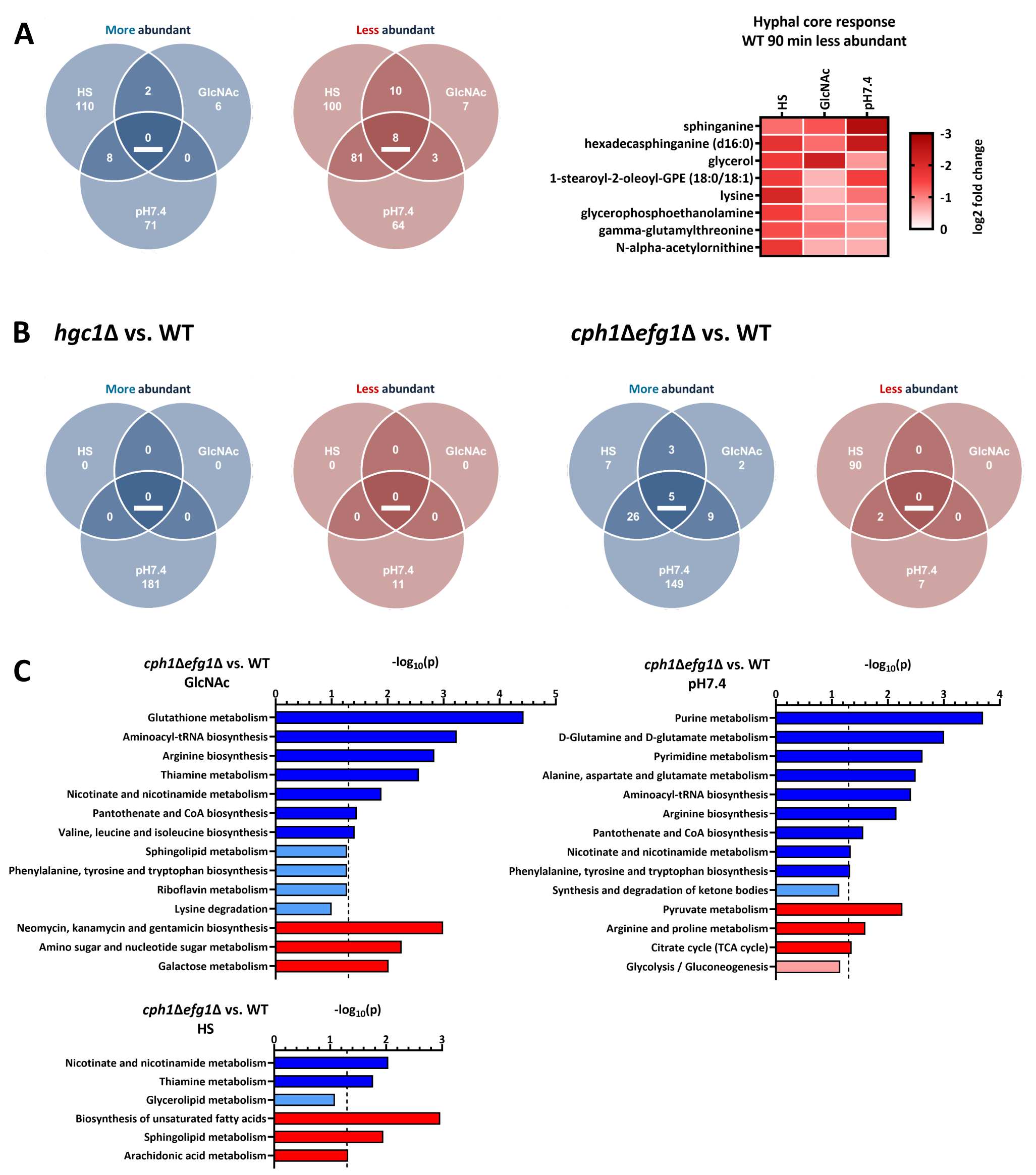

Supplement: FIG S5 [file msystems.00539-22-s0005.tif]

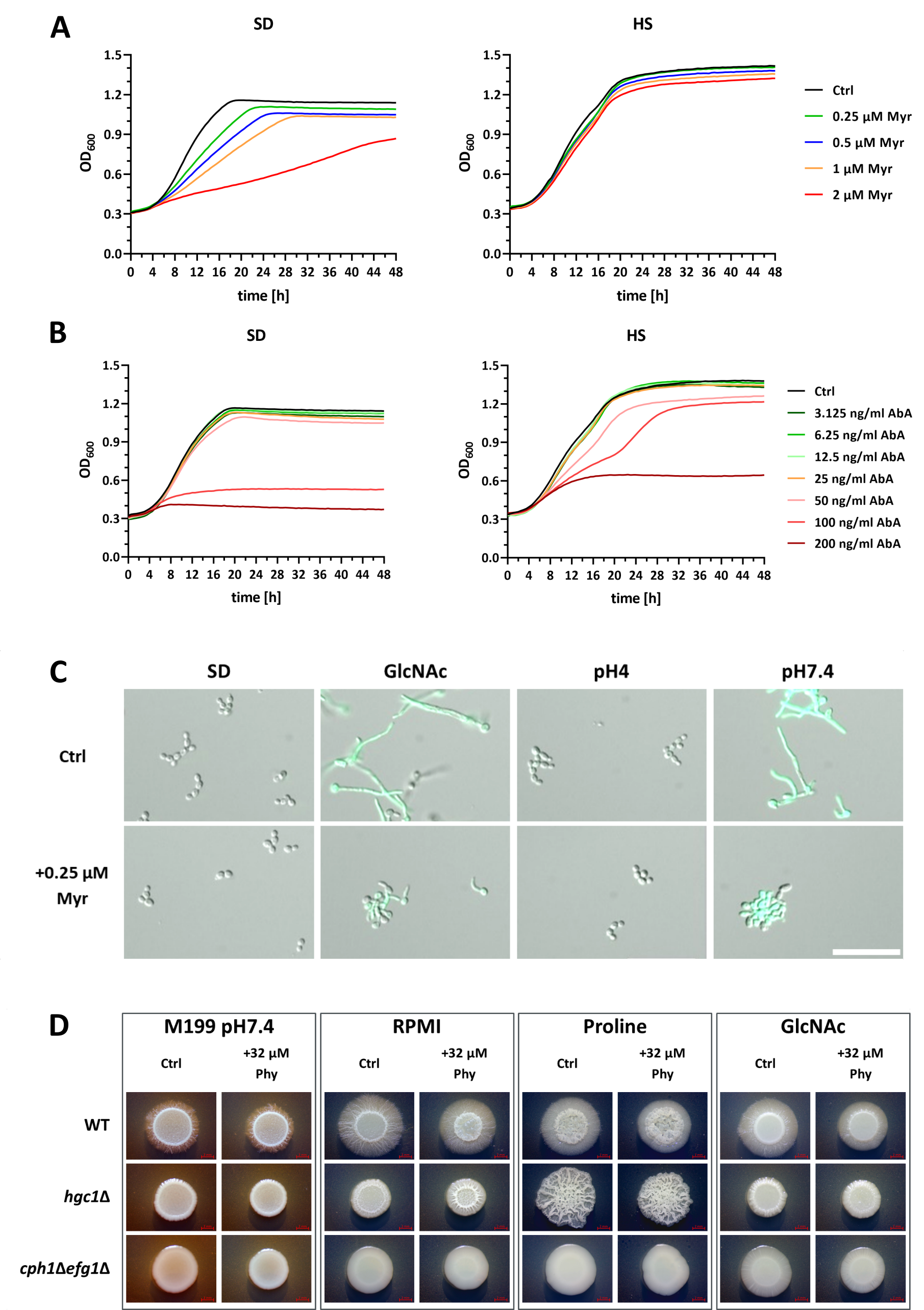

Supplement: FIG S6 [file msystems.00539-22-s0006.tif]
